# Towards a biocompatible artificial lung: Covalent functionalization of poly(4-methylpent-1-ene) (TPX) with *c*RGD pentapeptide

**DOI:** 10.3762/bjoc.9.33

**Published:** 2013-02-08

**Authors:** Lena Möller, Christian Hess, Jiří Paleček, Yi Su, Axel Haverich, Andreas Kirschning, Gerald Dräger

**Affiliations:** 1 Institut für Organische Chemie und Biomolekulares Wirkstoffzentrum (BMWZ) der Leibniz Universität Hannover, Schneiderberg 1B, 30167 Hannover, Germany; Fax: (+49) 511-762-3011; 2 Leibniz Research Laboratories for Biotechnology and Artificial Organs (LEBAO) Hannover Medical School (MHH), 30625 Hannover, Germany

**Keywords:** click chemistry, growth factor, nitrenes, plasma chemistry, poly(4-methylpent-1-ene), surface modification

## Abstract

Covalent multistep coating of poly(methylpentene), the membrane material in lung ventilators, by using a copper-free “click” approach with a modified cyclic RGD peptide, leads to a highly biocompatible poly(methylpentene) surface. The resulting modified membrane preserves the required excellent gas-flow properties while being densely seeded with lung endothelial cells.

## Introduction

Respiratory failures are a significant health-care problem with several hundred thousand adult patients each year [1]. Besides medical treatment, the use of mechanical ventilators that provide breathing support while the lungs recover, is often indispensable. This treatment is conducted when patients respond inadequately to medical therapy. However, invasive mechanical ventilation can damage the lungs physically by overpressurizing lung tissue or due to inflammation. This may lead to exacerbation of lung dysfunction or even to multiple-organ failure [2–3]. The morbidity and mortality associated with these problems still remains high.

**Figure 1:**
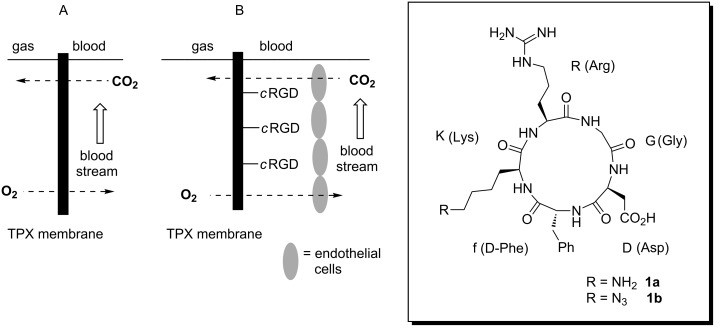
Schematic presentation of the artificial lung (A) and concept of membrane functionalization (B) with cyclic RGD **1b**.

Therefore, lung-support systems that perform the gas exchange extracorporeal can provide an alternative. They are connected to the patient via two cannulas in an arterio-venous circuit instead of an endotracheal aditus. These devices are based on polymeric hollow-fiber membranes that serve as an interface between blood and gas streams (Figure 1). The material must allow adequate gas exchange thus providing CO_2_ removal and O_2_ delivery for patients with respiratory or ventilatory failure. The thermoplastic polymer poly(4-methylpent-1-ene) (TPX; **2**) complies with this requirement. It is currently used as a polymeric hollow-fiber membrane material in lung-support systems due to its low density of 0.835 g/cm^3^ and therefore its high gas permeability, which allows an unhindered gas flow [4]. However, the artificial polymer surface leads to the formation of blood clots, which prevent long-term gas exchange and enforce the replacement of the device. This intervention is associated with undesired risks for the patient, such as bleeding and infections, as well as increased costs for the treatment.

Previously, experiments showed that the negative side effects of the artificial surface can be significantly reduced by seeding endothelial cells (ECs) onto a heparin/albumin-coated TPX surface [5]. Although these endothelialized membranes showed improved hemocompatibility, the cells were easily detached from the membrane due to the water-soluble protein coating, which is necessary for the cell attachment.

In this paper, we disclose a strategy to strongly attach ECs to TPX **2** membranes by covalent functionalization of the chemically rather inert material with a cyclic peptide, containing the RGD (arginine-glycine-asparagine) amino-acid sequence. We chose *c*RGD pentapeptide **1b** derived from lysine precursor **1a**, as *c*RGD’s intensively studied by the Kessler group [6–9] are well-established cell-recognition motifs that can trigger integrin-mediated cell adhesion [9]. Because of the high potential to stimulate this, these RGD peptides have successfully been used to generate biocompatible materials [10–11].

**Scheme 1:**
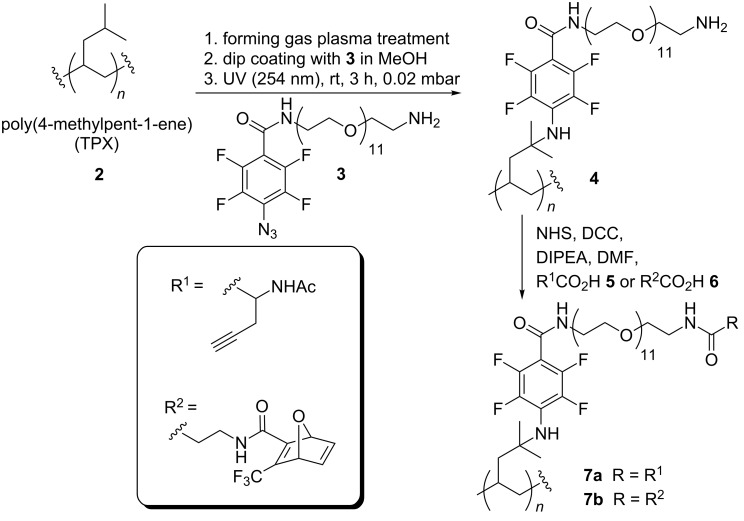
Functionalization of poly(4-methylpent-1-ene) (TPX) **2**.

Conceptually, we planned to first functionalize the hydrocarbon TPX **2** via UV-mediated nitrene insertion to form polymer derivative **4** (Scheme 1) [12–14]. Therefore, nitrene precursor **3** was modified with an oligo(ethylene glycol) linker, which served as a spacer unit between the polymer and the bioactive *c*RGD domain [15]. As the final coupling strategy, we chose the Huisgen-type azide–alkyne-“click”-chemistry for which connecting elements **5** and **6** were coupled to polymer derivative **4**. Classically, copper catalysts are required for these kinds of 1,3-dipolar cycloadditions, but their potential cytotoxic properties limit the usability in biomedical applications [16–17]. For overcoming this problem, several copper-free ligation methods were developed. In this work we pursue both options, i.e., the copper-mediated as well as the copper-free version [18].

## Results and Discussion

### Plasma-activation and dip coating of TXP

We found that chemical modification of the rather inert TPX membrane **2** was only possible in polar solvents because of the hydrophilic nature of the pegylated precursor **3**. Unfortunately, dip coating of TPX **2** with a methanolic solution of nitrene precursor **3**, and then drying followed by UV irradiation (Hg lamp, 254 nm) initially did not lead to surface modification. The presence of F and N on the TPX membrane was not detectable by XPS analysis. The photoinduced reaction of azide **3** with cyclohexane served as a model reaction and provided the expected tetrafluoro-substituted arylcyclohexyl amine in 47% yield, proving the viability of the functionalization concept (see Supporting Information File 1, section 2D). We assumed that the hydrophobic nature of TPX **2** inhibited the bedabbling of the polymer with linker **3**. Therefore, TPX **2** was pretreated with different types of plasma under conditions that resulted in lower contact angles (see below). We chose atmospheric-pressure plasma, which simplifies the procedure because the plasma chamber does not need to be evacuated [19]. Importantly, the plasma had to be optimized (with respect to the gas composition, time of treatment, distance of the plasma to the polymer surface, and electronic parameters; see Supporting Information File 1) in order not to damage the membrane by creating holes or altering the shape of the material. This optimization finally resulted in the use of a low-energy forming-gas plasma (10% H_2_). The use of plasma for altering the surface properties of polymers is well established. It can be expected that only the surface is partially oxidized without affecting the integrity of the material [20–22].

Next, the TPX membrane **2** (see below; bars are given in cm) was treated with a solution of azide **3** as described above and UV-irradiation gave covalently functionalized polymer **4**. For practicability reasons, flat TPX membranes instead of hollow fibers were used.

### Physicochemical analysis of modified TPX foils

**Figure 2:**
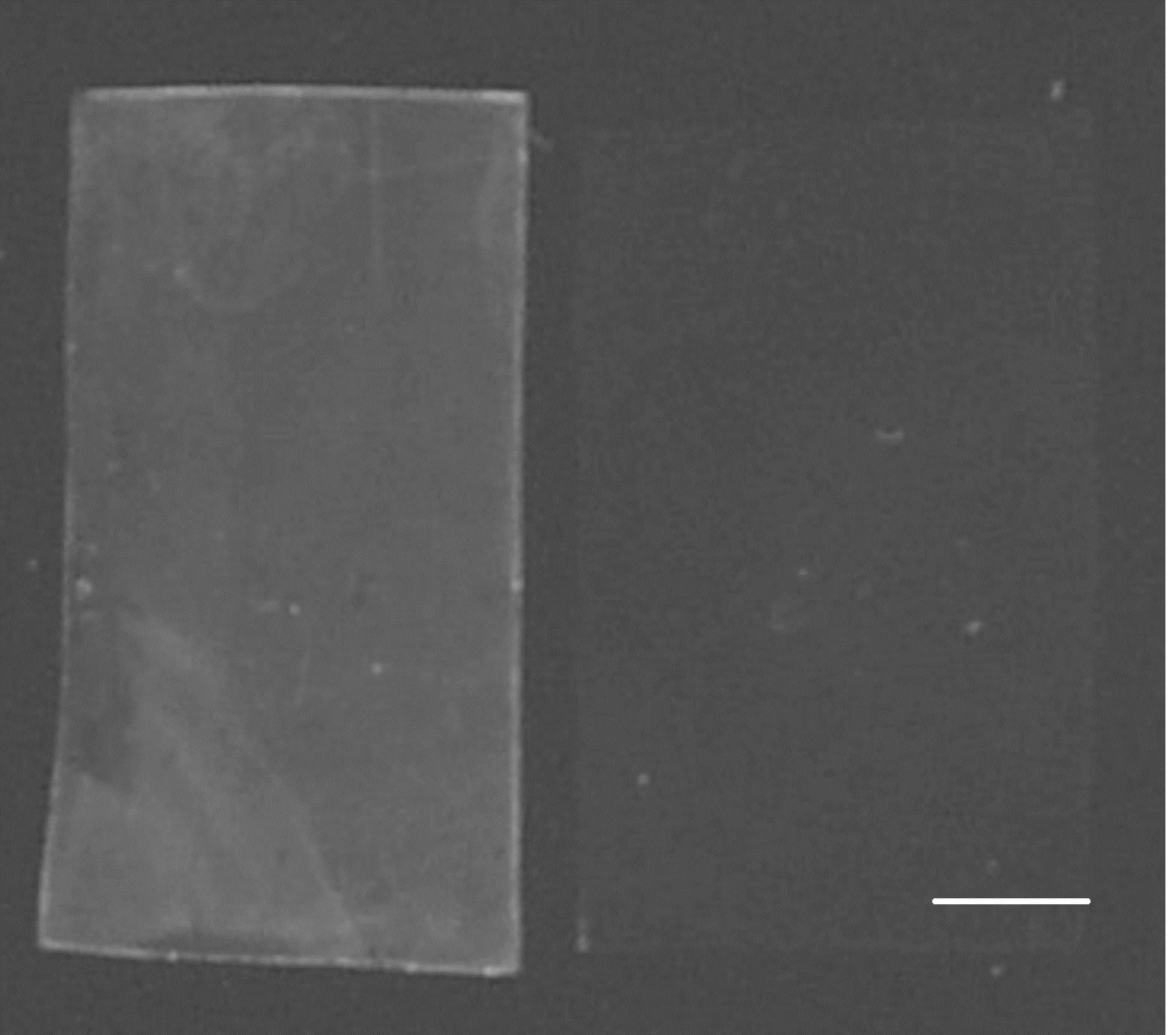
Analyses of functionalized TPX membranes with UV light at 312 nm; left: derivative **4**; right: nonfunctionalized material as negative control (scale bar equals 1 cm).

Analysis of all materials, including starting material TPX **2**, was achieved with different methods described in the following. Contact-angle measurements showed an increase of about 60% compared to the plasma-treated material, which is a diagnostic for the coverage of the surface with linker **3**. The IR spectrum of **4** displayed carbonyl vibrations at 1640 cm^−1^. The IR vibration for the azido moiety at a wavelength of 2120 cm^−1^ was absent, indicating that no adsorbed linker molecules were present on the TPX surface (see Supporting Information File 1, Figure S1). Additionally, UV served to prove successful CH insertion of the nitrene moiety. Irradiation with UV light at 312 nm clearly showed absorption generated from the π-system of the fluorinated phenyl ring (Figure 2). In contrast to this, nonfunctionalized foils did not show any absorption.

**Figure 3:**
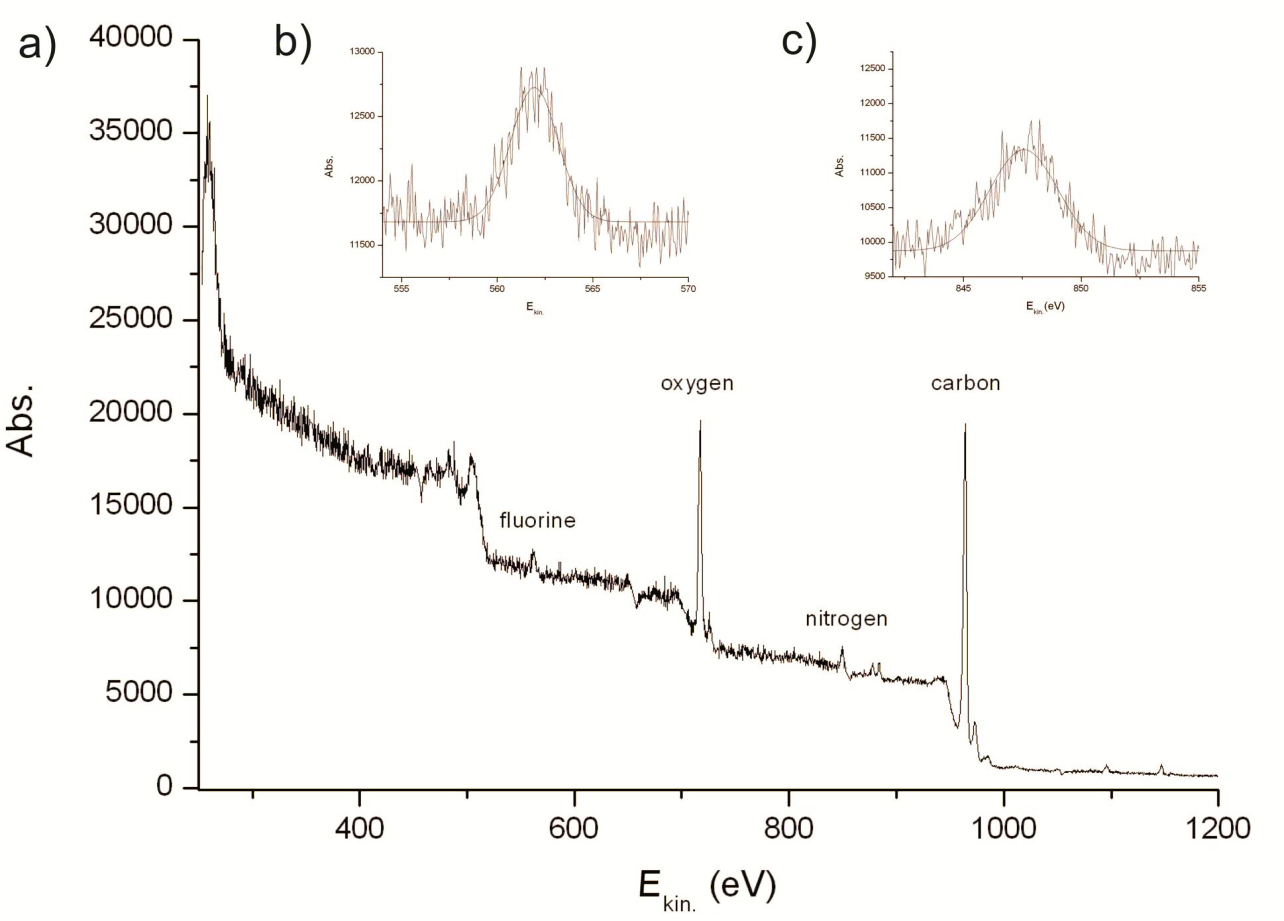
Analyses of functionalized TPX 4 by XPS spectroscopy using *h*ν = 1250 eV; (a) overview, (b) F 1s core level, (c) N 1s core level.

X-ray photoelectron spectroscopy (XPS) analysis of functionalized TPX membrane **4** was used to determine the elements on the polymer surface (Figure 3a). The strongest peak with a kinetic energy of 967 eV was assigned to the 1s core level of the carbon polymer backbone. In addition to carbon, oxygen (O 1s at *E*_kin._= 720 eV) could be monitored on the surface, which can be ascribed to the oxidative pretreatment with the forming-gas plasma. Fluorine and nitrogen were also detected on TPX derivative **4** (Figure 3b and 3c) by XPS measurements. The fluorine peak (F 1s at *E*_kin._= 562 eV) was undoubtedly related to the inserted linker because unmodified TPX membranes did not show any absorption at 562 eV. Nitrogen (N 1s at *E*_kin._= 846 eV) was already present on the surface after plasma pretreatment but its percentage significantly increased after the insertion process. Importantly, gas permeability tests showed that the chemical modification of TPX did not alter its excellent gas-flow properties (see Supporting Information File 1, section 8). These analyses clearly demonstrate that plasma treatment prior to the reaction with nitrene from precursor **3** provides the hydrophilization of the polymer, which is a prerequisite for successful dip-coat functionalization.

**Scheme 2:**
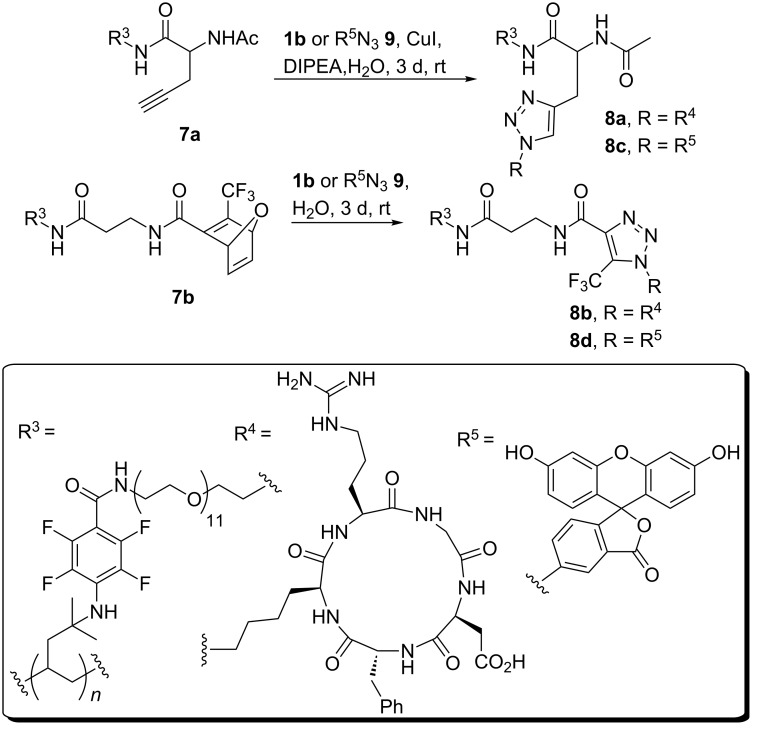
Copper-catalyzed and copper-free azide–alkyne “click“ reaction between functionalized TPX membranes **7a** or **7b** and *c*RGD peptide **1b** or with fluorescein R^5^N3 **9** for analytic purposes.

With these results on functionalized TPX **4** in hand we continued the synthesis towards RGD functionalized TPX. Therefore, simple washing protocols for facile workup were applied because all reactions were performed on the polymer surface. Thus, TPX derivative **4** was either coupled with alkyne **5** or with oxa-norbornadiene **6**, by using classical coupling chemistry, which resulted in polymers **7a** and **7b**, respectively (Scheme 1). The alkyne-containing amino acid **5** was prepared according to Brea et al. [15] Membrane **7a**, synthesized from polymer **4** and amino acid **5**, was coupled with RGD peptide **1b**, bearing an azido group, by the use of Huisgen-type “click” chemistry (Scheme 2). *c*RGD pentapeptide **1b** was prepared in sufficient amounts by solution-phase chemistry [23]. Because of the disadvantage associated with copper-mediated 1,3-cycloadditions of alkynes with azides in biological or biomedical applications we alternatively pursued a copper-free approach that relied on the oxanorbornadiene strategy of Rutjes [24–28]. This type of specific conjugation most likely proceeds by a 1,3-dipolar cycloaddition/retro-Diels–Alder cascade. By incubating oxanorbornadiene functionalized membranes **7b** with *c*RGD pentapeptide **1b** the cycloaddition product **8a** was formed in the absence of any additives (Scheme 2). Prior to chemical reactions carried out with modified TPX materials, all reactions were first probed in solution (see Supporting Information File 1, section 2D).

**Figure 4:**
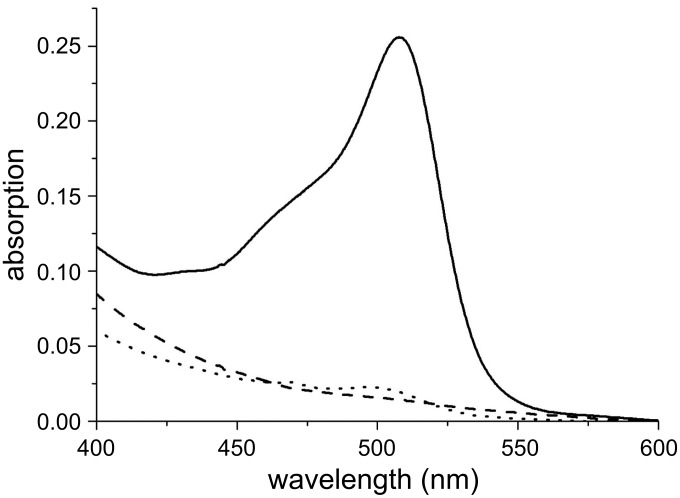
UV spectra of TPX **8c** and **8d** functionalized with fluorescein revealing the efficiency of the 1,3-dipolar cycloaddition reactions on modified TPX membranes. Continuous line: **8d**; dotted line: **8c**; dashed line: substrate for Huisgen-type “click” reaction **7a**.

In order to analyze the outcome of 1,3-dipolar cycloadditions, the model compound fluoresceinyl azide **9**[29–30] was first coupled to TPX derivatives **7a** or **7b**, under identical conditions as described for *c*RGD peptide **1b**. The resulting polymers **8c** and **8d** were studied by determining the UV absorption peaks of fluorescein between 400 and 600 nm (Figure 4). Absorption maxima of fluorescein were located at 457 nm and 481 nm, which can be ascribed to the presence of two isomeric forms (lacton versus carboxylate) of fluorescein [31]. These measurements clearly revealed the successful covalent functionalization of TPX with fluorescein by 1,3-dipolar cycloaddition. The copper-catalyzed method provided material **8c**, which showed a small but significant absorption maximum for fluorescein at about 515 nm (dotted line versus dashed line) compared to starting TPX **7a**.

In contrast, the copper-free method led to intense fluorescein staining (polymer **8d**, continuous line) of the TPX membrane with an increased factor for the absorption intensity of about 65 compared to **8c**. This remarkable result may be rationalized if one assumes that copper is not ideally distributed during the course of the 1,3-dipolar cycloaddition reaction, as the presence of the heterogeneous TPX foils hampers continuous stirring. When functionalized polymers **8c** and **8d** were washed by soxhlet treatment (six hours in methanol) no change of the color of the polymer membranes or the extracts was encountered. It must be noted that neither TPX **2** nor functionalized analogues **4**, **7a** or **7b** have an absorption maximum between 400 and 600 nm. In addition, plasma-treated unfunctionalized TPX membrane **2**, lacking an alkyne functionality, was treated with azide **9** in order to determine the degree of physisorption of fluorescein. Washing of the membrane, as routinely done in this study, provided a material that did not reveal absorption maxima between 440–600 nm. These results demonstrate that the 1,3-dipolar cycloaddition protocols gave covalently linked fluorescein–TPX membrane adducts.

### Endothelialization of TPX foils

Based on these promising results, we next tested the biological properties of the new TPX materials **8a** and **8b** functionalized with *c*RGD. Particularly, we investigated the growth of endothelial cells (ECs) through integrin-mediated binding. ECs were seeded at a density of 1 × 10^4^ cells/cm^2^ on TPX membranes **8a** and **8b** [5]. Adherence, growth and viability of cells were monitored and quantified by calcein staining and fluorescence imaging (see Supporting Information File 1, section 2B).

Unmodified TPX membranes were used as negative control, while albumin/heparin coated TPX, which are currently used in clinical applications, were applied to monitor cell viability. After 48 h cultivation the formation of a confluent endothelial cell monolayer was observed on both the *c*RGD as well as albumin/heparin-modified membranes. In contrast, no adherent cells could be found on the unmodified membranes **2**. To verify that the observed effect is in fact only RGD-mediated, membranes obtained during every functionalization step en route to RGD-modified polymers **8a** and **8b** were also analyzed. We found that seeding of cells onto the TPX membranes that were treated with forming-gas plasma gave cell coverage comparable to the control with heparin/albumin. This effect is associated with increased hydrophilicity resulting from the oxidative plasma conditioning on the surface [32]. The plasma-mediated surface oxidation yields polar groups including hydroxy groups, which rapidly vanish, a well-investigated aging effect of plasma-treated materials. This process is an entropy-initiated surface reorientation and it hampers direct chemical modification of these oxidized surfaces [32]. Also our TPX membrane **2** revealed this aging effect, as judged by changes of the contact angles. Plasma treatment led to a lower angle (from 107°± 1° to 46° ± 7°), which increased to 70° ± 5° after one week and further to 75° + 2° within two months, making plasma-treated TPX **2** not suited for biomedical applications.

**Figure 5:**
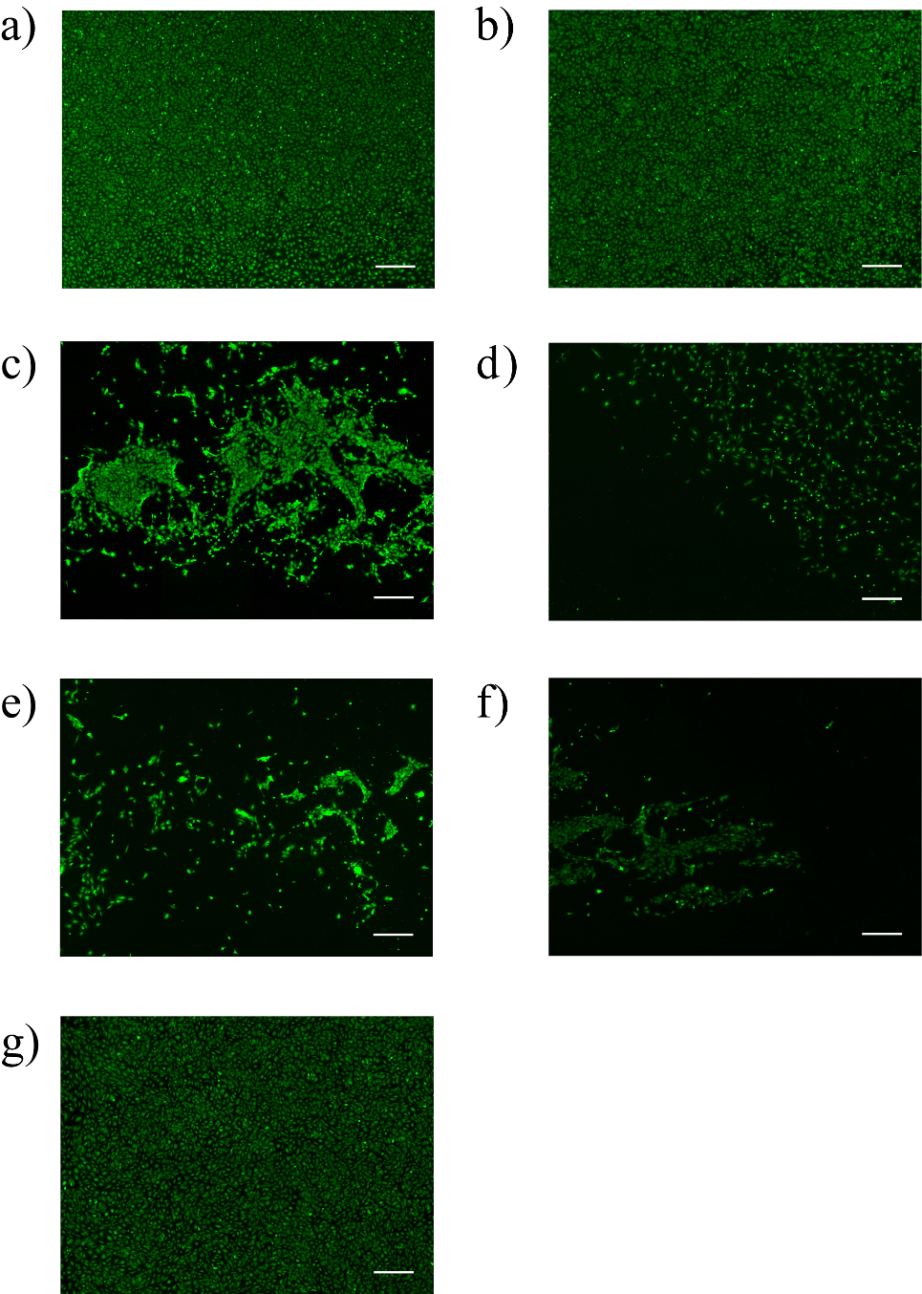
Cell seeding onto TPX derivatives (scale bar equals 500 µm) (a) **2** as control with heparin/albumin dip coating; (b) **2** treated with forming-gas plasma; (c) functionalized TPX **4** with PEG; (d) functionalized TPX **7a** with PEG and alkyne group; (e) functionalized TPX **8a** with *c*RGD (copper-catalyzed approach); (f) functionalized TPX **7b** with PEG and oxanorbonadiene group; (g) functionalized TPX **8b** with *c*RGD (copper-free approach).

Once the TPX-membrane was functionalized with the PEG unit, a significant reduction of the number of adhered cells was encountered (Figure 5c). This effect was even more pronounced on TPX membranes **7a** and **7b**, where the ECs were either exposed to the alkyne or the oxanorbornadiene-functionalized surfaces, respectively (Figure 5d and 5f). This observation correlates well with previous reports showing that cells do not attach on PEGylated polymers as long as no additional growth factors are present [33]. Finally, TPX surfaces **8a** and **8b** functionalized with *c*RGD behaved differently regarding their cell-adhesion properties (Figure 5e and 5g). These tests strongly indicate that copper-catalyzed attachment of cRGD pentapeptides only provides a minor increase of biocompatibility, despite the fact that the functionalized TPX **8a** was excessively treated with an aqueous solution of EDTA in order to remove all copper traces adsorbed on the polymer. In contrast, TPX membranes modified under copper-free “click” conditions were homogenously covered with endothelial cells (Figure 5g). This difference in surface coverage of ECs can be rationalized either by (a) either the toxicity of the remaining copper traces or (b) by the lower amount of surface-bound *c*RGD peptide on the TPX membrane **8a** (see Figure 4). It is worth noting that the experiments were repeated eight times and revealed high reproducibility of biological response for a given mode of chemical modification.

Finally, we also repeated the whole synthetic sequence, but this time using a PEG unit with a molar-mass distribution of 3000 g/mol instead of the defined PEG linker **3**. This modified material should reveal the impact of linker length on the effectivity to grow ECs on TPX membranes. We found that this new TPX membrane **8a** showed reduced biological potency to bind ECs. This observation is in accordance with studies by Beer et al. who showed that virtually all RGD binding sites can be reached by integrins, when the distance between the surface and the RGD peptide amounts to 46 Å. Access by integrins again decreases when the spacer is longer and likely folds in such a way that RGDs become less surface-exposed [34].

## Conclusion

In conclusion we developed a protocol for functionalizing polyhydrocarbons, here poly(4-methylpent-1-ene) (TPX), which are important biomedical materials. For this purpose, nitrene insertion proved to be a powerful way of achieving this functionalization, but only after the polymer had undergone plasma pretreatment. Further derivatization was achieved that allowed the introduction of *c*RGD pentapeptides by using either copper-catalyzed or copper-free “click” protocols on TPX surfaces. Finally, *c*RGD-functionalized TPX membrane surfaces showed excellent biocompatibility regarding the adhesion of endothelial cells. These studies pave the way for the development of improved, extracorporeal oxygenators.

We believe that the combination of plasma treatment with nitrene insertion is a protocol of general importance for the functionalization of biomedical materials based on hydrocarbon-derived polymers. The importance of developing strategies for this kind of copper-free surface functionalization reported here was recently also demonstrated by the Lahann group [35]. We believe that the copper-free approach described is of general importance and should be transferrable to (bio)polymers, inorganic materials or to metals such as titanium used in biomedical devices [36].

## Supporting Information

Details on the synthesis and analyses of building blocks and linkers, functionalization, biological evaluation and descriptions of analyses of TPX materials based on XPS, UV, IR and contact-angle measurements are found in Supporting Information File 1.

File 1 Experimental and analytical data.
