# Supplementary material for: Towards a biocompatible artificial lung: Covalent functionalization of poly(4-methylpent-1-ene) (TPX) with cRGD pentapeptide
Source: Beilstein J Org Chem. 2013 Feb 8;9:270–7. doi: 10.3762/bjoc.9.33 (PMC3596089; doi:10.3762/bjoc.9.33)
Supplement: File 1 — Experimental and analytical data. [file Beilstein_J_Org_Chem-09-270-s001.pdf]

Supporting Information

for

Towards a biocompatible artificial lung: Covalent functionalization of poly(4-methylpent-1-ene) (TPX) with *c*RGD pentapeptide

Lena Möller[1], Christian Hess[2], Jiří Paleček[1], Yi Su[1], Axel Haverich[2], Andreas Kirschning*[1] and Gerald Dräger*[1]

Address: [1]Institut für Organische Chemie und Biomolekulares Wirkstoffzentrum (BMWZ) der Leibniz Universität Hannover, Schneiderberg 1B, 30167 Hannover, Germany; Fax: (+49) 511-762-3011 and [2]Leibniz Research Laboratories for Biotechnology and Artificial Organs (LEBAO) Hannover Medical School (MHH), 30625 Hannover (Germany)

Email: Gerald Dräger - gerald.draeger@oci.uni-hannover.de; Andreas Kirschning - andreas.kirschning@oci.uni-hannover.de

* Corresponding author

Experimental and analytical data.

Content

1. Overview: Syntheses

2. Experimental part

2A. General information

2B. Biological evaluation

2C. Syntheses

2D. Model reactions

3. Analysis of the intrene insertion with ATR-FT-IR

4. High-resolution XPS spectra of plasma-treated TPX membranes and plasma-treated and -modified TPX membranes **4**

5. Quantification of the linker on the polymer surface

6. UV spectra of Cu-catalysed “click” chemistry

6A. Fluorescein coupled TPX derivatives with fluorescein and negative control

6B. Fluorescein coupled by Cu-free “click” chemistry

7. Contact angles

8. Measurements of carbon dioxide and oxygen permeability through TPX and functionalized TPX membranes

9. NMR spectra

10. References

1 Overview: Syntheses

a) The synthesis of the fluorinated phenyl azide suitable for CH-insertion on the polymer (synthesis of azide **S3** was carried out according to Keana et al. [1]):

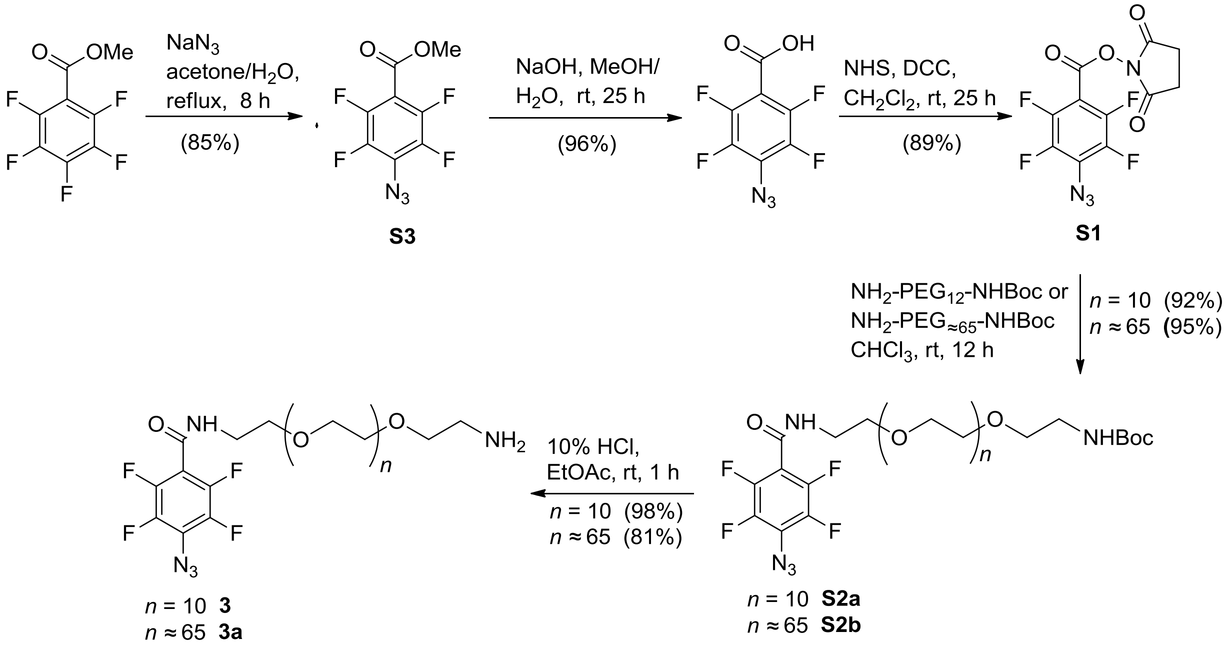

Modification of TPX with PFPA-$PEG_{\approx 65}$-NH_2 was performed in the same way as described for PFPA-PEG_{11}-NH_2.

b) Synthesis of the alkyne extension unit according to Brea et al. [2]:

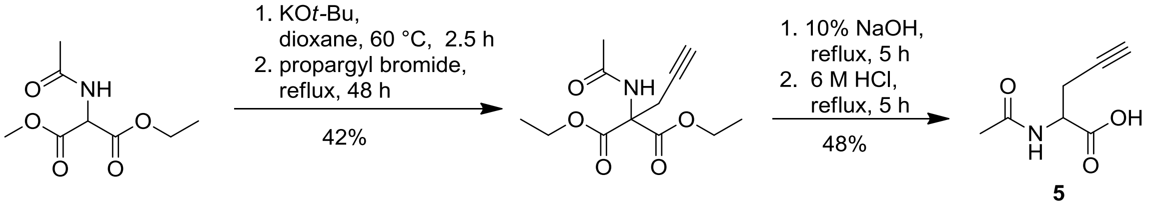

c) Synthesis of fluoresceinyl azide according to Molnar et al. [3]:

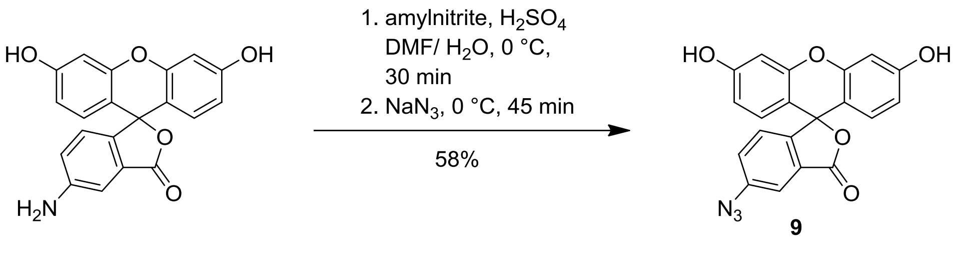

d) Synthesis of the oxanordornadiene linker for copper-free click chemistry (according to Su et al. [4] , based on Rutjes et al. [5]:

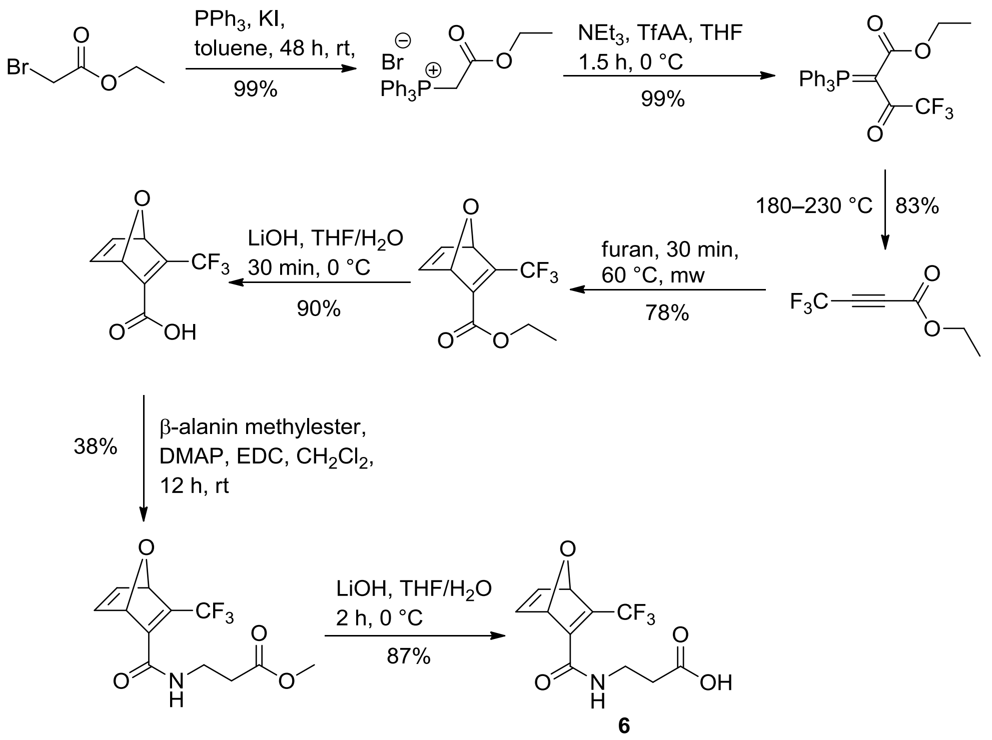

e) Modification of the polymer (copper-catalyzed ligation):

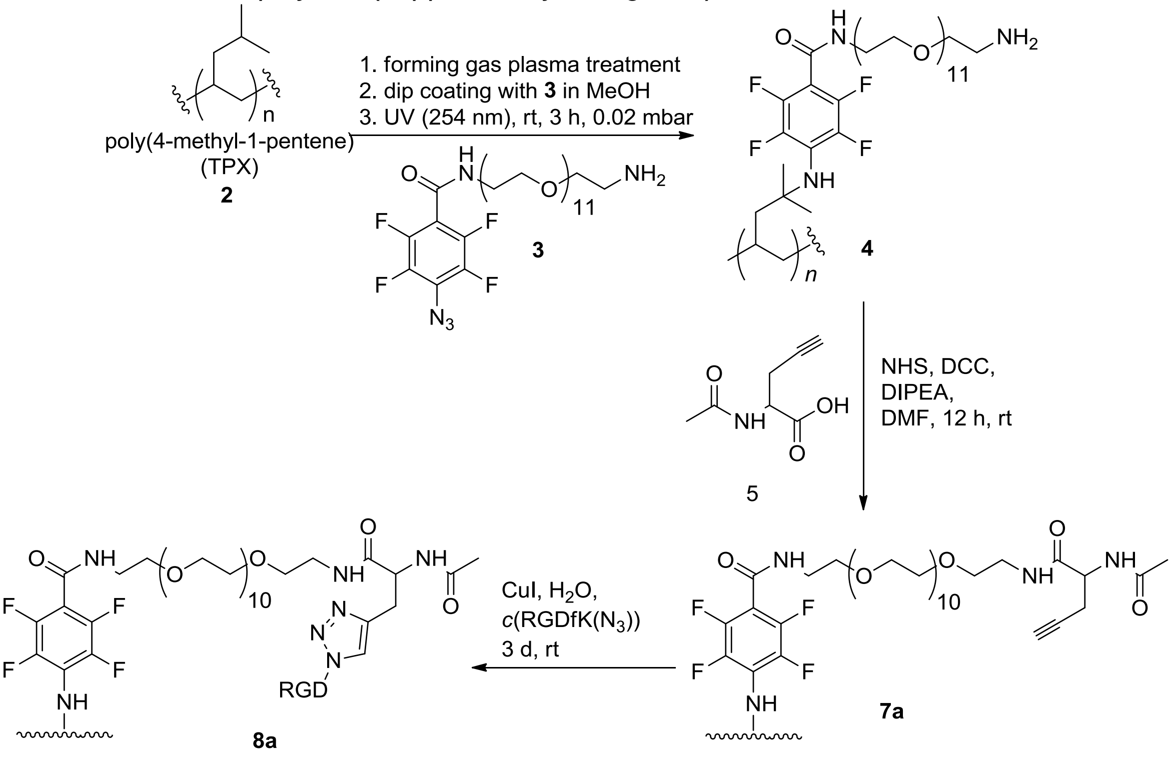

f) Modification of the polymer (copper-free ligation):

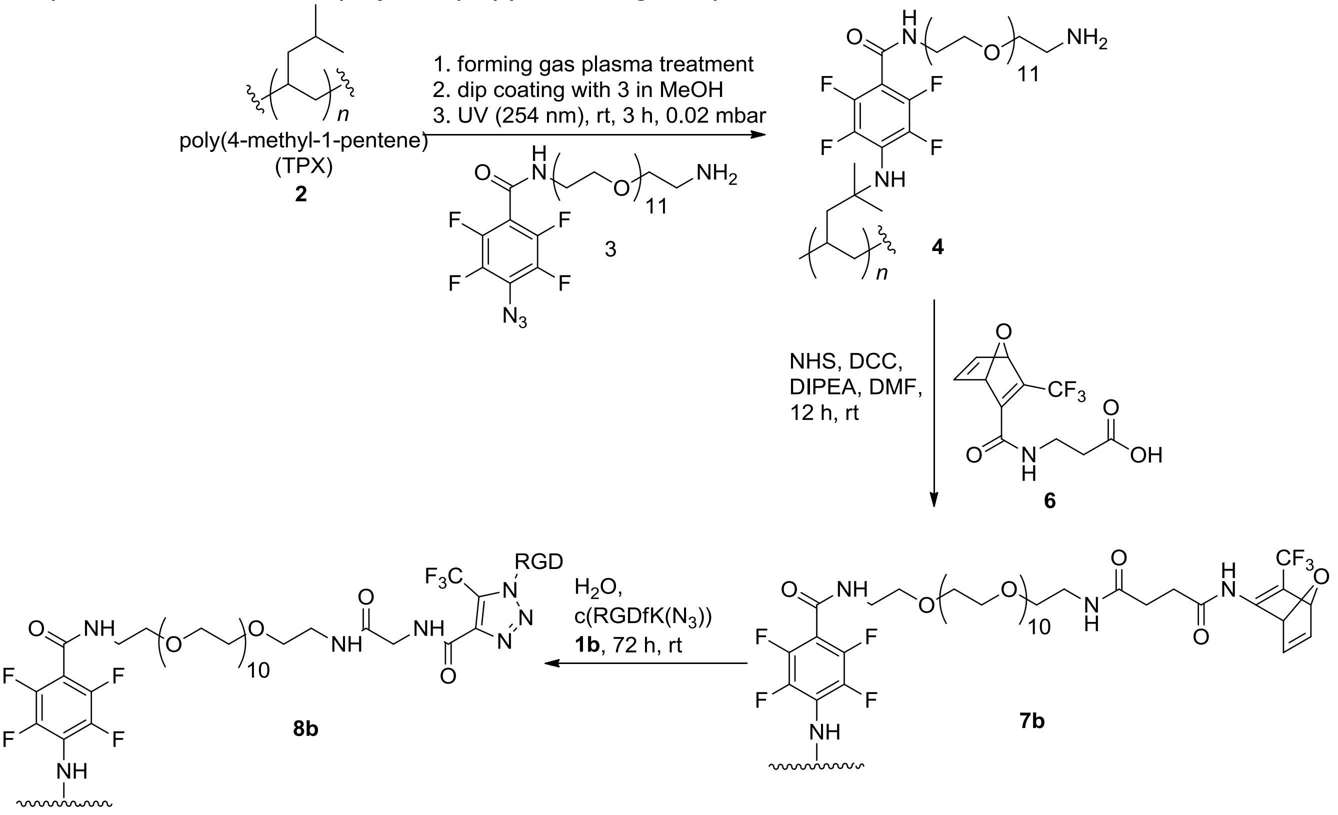

2. Experimental part

2A. General information

Unless otherwise stated, all chemicals and solvents were purchased in p.a. quality from *Sigma-Aldrich* or *Acros Organics*, Geel (Belgium) and used as received. Poly(4-methyl-1-pentene) (TPX) membranes (thickness 30µm) were purchased from *Goodfellow*, Bad Nauheim (Germany).

Analytical thin-layer chromatography was performed using precoated silica gel 60 F254 plates (*Merck*, Darmstadt, Germany), and the spots were visualized with UV light at 254 nm or with different staining reagents and addition of heat. Flash column chromatography was performed on Merck silica gel 60 (230-400 mesh) under slight pressure. When size-dependent purification was necessary, Sephadex® LH-20 silica gel was used with methanol as eluent. HPLC purification was performed with a preparative HPLC device *Merck Hitachi* LaChrom (pump: L-7150, Interace D-7000, Diode-Array-detector L-7450 {λ = 220–400 nm}). C-18-$P_{[A]}$Trentec Reprosil 120 C 18 AQ (5 mm, 250 mm diameter: 25 mm) was used as stationary phase.

All ^{1}H NMR spectra were recorded at room temperature using a DPX-200 (200 MHz), Avance-400 or DPX-400 (400 MHz) spectrometer from *Bruker*, Karlsruhe (Germany) in solvents obtained from *Deutero*, Kastellaun (Germany). Remaining signals from nondeuterated solvents were used as standards. ^{13}C NMR spectra were measured with the devices Avance-400 or DPX-400 (100 MHz) from *Bruker.* Mass spectra were recorded with a type LCT (ESI) from Micromass, Manchester (UK) in methanol or acetonitrile.

Absorption of UV-light was measured with a *Shimadzu,* Duisburg (Germany) UV-1601PC spectrophotometer. Hydrophilic/hydrophobic nature of either uncoated or coated TPX membranes was determined by contact angle measurements with a SURFTENS UNIVERSAL contact-angle meter (*OEG,* Frankfurt/Oder) using the sessile drop method under ambient laboratory conditions. Therefore, three drops of distilled water were placed on the surface of the TPX foils and their contact angle was averaged.

Melting points were determined with an optimelt device from *SRS*, Sunnyvale (USA). IR measurements were performed with an ATR-FT-IR spectrometer (vector 22) from *Bruker*. Combustional analyses were carried out with a Vario EL device from *Elementar Analysensysteme*.

As UV light source for insertion reactions, which emits light at 254 nm, a mercury low-pressure immersion lamp TNN 15/32 with a capacity of 15 W and a voltage of 55 V was used.

2B. Biological evaluation

Heparinized human umbilical cord blood (25 I.E. heparin/ml), obtained from healthy newborn donors after informed parental consent, was mixed 1:1 with PBS. Ten milliliters from each donor was subjected to density gradient centrifugation on 15 ml Ficoll-Hypaque for 20 min at 800 × *g* at 20 °C without brake. The interphase containing mononuclear cells (MNC) was collected, washed with 35 ml PBS and centrifuged for 20 min at 400 × *g*. MNCs were seeded on 1% gelatin precoated plastic plates at a density of 10^5 cells/cm^2. Cells were cultured in endothelial growth medium (Endothelial cell basal medium (EBM-2, *Lonza, Verviers*) with 10% FCS and growth factor supplements (EGM-2) containing VEGF, EGF, FGF-2, R3-IGF-1, hydrocortisone, ascorbic acid, heparin, penicillin / streptomycin). Culture plates were scored for the presence of endothelial cell colonies starting 5 days after the onset of culture. With the appearance of adherent endothelial cell clusters with cobblestone-like morphology at week 1-2 of culture, cells were adapted to EGM-2 containing 2% FCS. Every 48 h, 2/3 of the medium was replaced by fresh EGM-2. Reaching 70% confluence, human CB-ECFCs were trypsinized and reseeded at a density of 0.8×10^3 cells/cm^2. The endothelial phenotype of cells was verified using flow cytometry and RT-PCR as described before by Hess et al. [6].

Square pieces (6 cm^2) of membranes from every functionalization step were seeded with ECFCs at a density of 3.5×10^4 cells/cm^2. Uncoated and albumin/heparin-coated TPX membranes were used as negative and positive controls, respectively. Cells were further cultivated in EGM-2. After 2 days the cells were stained with calcein-acetomethylester (calcein-AM; stock solution 1mg/ml in DMSO; working concentration 5μg/ml; Molecular probes, *Eugene*, USA) for 30 min at 37 °C. Subsequently, adherence and growth of cells was monitored by fluorescence imaging (Axio Observer A1 microscope).

2C. Syntheses

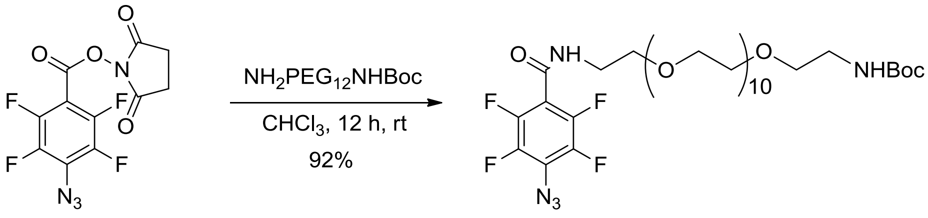

S1 — $C_{11}H_4F_4N_4O_4$, 332,10 g/mol

S2a — $C_{36}H_{59}F_4N_5O_{14}$, 861.40 g/mol

NH_2-PEG_{12}-NHBoc (0.265 g, 412.5 μmol, 1.0 equiv) and 4-azidotetrafluoro-benzoic acid-*N*-succinimidylester **S1** (0.164 g, 0.495 mmol, 1.2 equiv) were dissolved in chloroform and stirred for 12 hours at room temperature. The solvent was removed in vacuo and the crude product was purified by column chromatography on Sephadex (eluation with methanol).The product **S2a** was obtained (326.9 mg, 380.4 μmol; 92%) as a colorless oil.
DC: R_F (dichlormethane / methanol 95/5) = 0.21, ^{1}H NMR (400 MHz, $CDCl_3$) δ 1.43 (s, 9 H, 3 × CH_3^{Boc}), 3.15–3.96 (m, 48 H, CH_2^{PEG}), 5.04 (s(br), 1 H, N*H*Boc), 6.96 (s, 1 H, N*H*COAr) ppm; ^{13}C NMR (100 MHz, $CDCl_3$) δ 27.53 (3 × CH_3^{Boc}), 39.18 ($C^{quart.Boc}$), 49.86 (2 × CH_2NH), 68.50–69.69 (CH_2^{PEG}), 111.08 ($C^{arom.}$CO), 120.61 ($C^{arom.}N_3$), 138.17, 140.63, 141,82, 144.50 ($C^{arom.}$F), 155.15, 156.90 (CO^{PFPA}, CO^{Boc}) ppm; HRMS: $C_{36}H_{59}F_4N_5NaO_{13}$ $[M + Na]^+$ calcd for 884.3892, found: 884.3885.

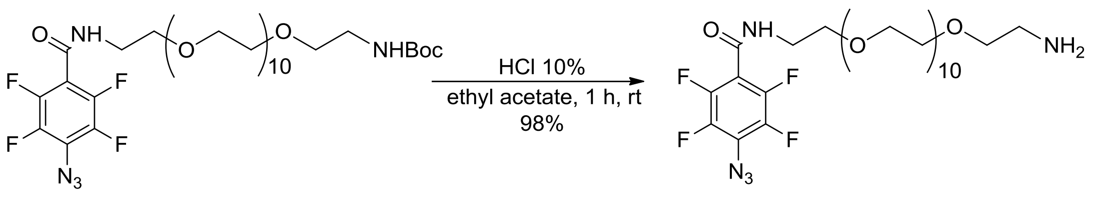

S2a **3**

$C_{36}H_{59}F_4N_5O_{14}$
862.00 g/mol

$C_{31}H_{51}F_4N_5O_{12}$
761.35 g/mol

10% Aqueous hydrochloric acid (10 mL) was slowly added to **S2a** (666.2 mg, 772.85 µmol, 1.0 equiv) in ethyl acetate (15 mL) and stirred for 1 hour at room temperature. The solvent was removed in vacuo to afford product **3** (0.76 mmol; 98%) as a colorless oil.
^{1}H NMR (400 MHz, $CDCl_3$) δ 3.19–3.87 (m, 48 H, CH_2^{PEG}), 6.26 (s, 1 H, N*H*), 7.89 (s(br), 2 H, NH_2) ppm; ^{13}C NMR (100 MHz, $CDCl_3$) δ 40.31, 40.73 (*C*H_2NH, *C*H_2NH_2), 67.20–70.71 (*C*H_2^{PEG}), 112.67 ($C^{arom.}$CO), 121.62 ($C^{arom.}N_3$), 139.44, 142.10, 143.09, 145.59 ($C^{arom.}$F), 158.54 (*C*ONH) ppm, HR-MS: $C_{31}H_{52}F_4N_5O_{12}$ $[M+H]^+$ calcd for 762.3549, found: 762.3550.

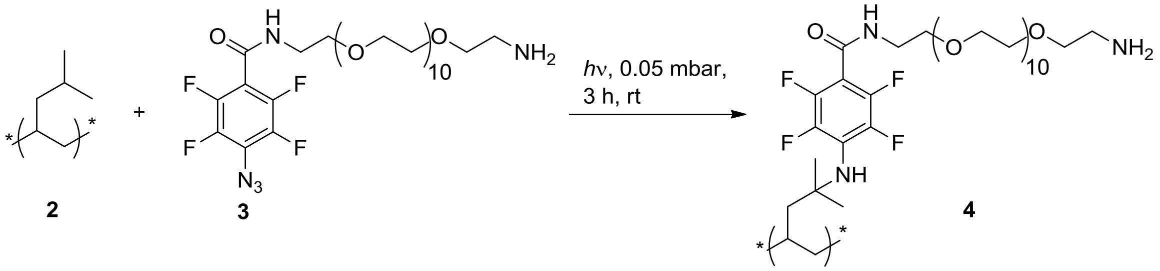

2 **3** **4**

$C_{31}H_{51}F_4N_5O_{12}$
761.35 g/mol

The plasma-treated or alternatively the non-plasma-treated TPX foils **2** were soaked in a solution of linker **3** in methanol (*c* = 10 mg/mL) for 12 hours at room temperature. The moistened membranes were dried in vacuo and placed into quartz glass flasks. Subsequently, they were exposed for 3 hours to a low-pressure mercury lamp, while the quartz glass setup was still evacuated to a pressure of 0.05 mbar. The foils were rotated several times during the exposure process.
Afterwards, the foils were washed with demineralized water, isopropanol and methanol and dried in vacuo.
The analysis of the decorated foils was performed as described in the main manuscript and in sections 3-5 (see below). The insertion of the linker with approximately 65 PEG units was performed in the same way as described for **4**.

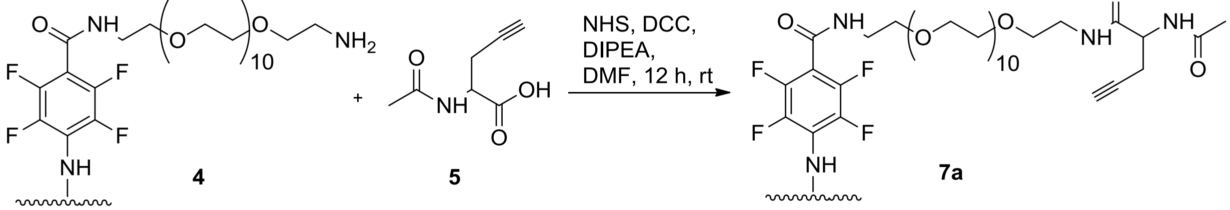

4 **5** **7a**

$C_7H_9NO_3$
155.06 g/mol

2-Acetaminopent-4-ynoic acid (**5**, 20.5 mg, 131.35 µmol, 1.0 equiv), *N*-hydroxysuccinimide (30.43 mg, 264.41 µmol, 2.0 equiv), dicyclohexyl carbodiimde (54.56 mg, 264.41 µmol, 2.0 equiv), and diisopro-

pylethylamine (112.42 µL, 661.10 µmol, 5.0 equiv) were dissolved in dimethylformamide (30 mL) under argon atmosphere. The TPX foils were added and the reaction mixture was stirred at room temperature for 12 h.
Afterwards, the foils were washed with demineralized water, isopropanol and methanol and dried in vacuo.
The modified foils **7a** were not analyzed directly but the reaction between linker **3** and 2-acetamino-pent-4-yloic acid **5** served as a model reaction in order to analyze the best coupling conditions (see chapter 2d below). The amide coupling of the TPX derivative with the approximately 65 PEG units longer linker was performed in the same way as described for **7a**.

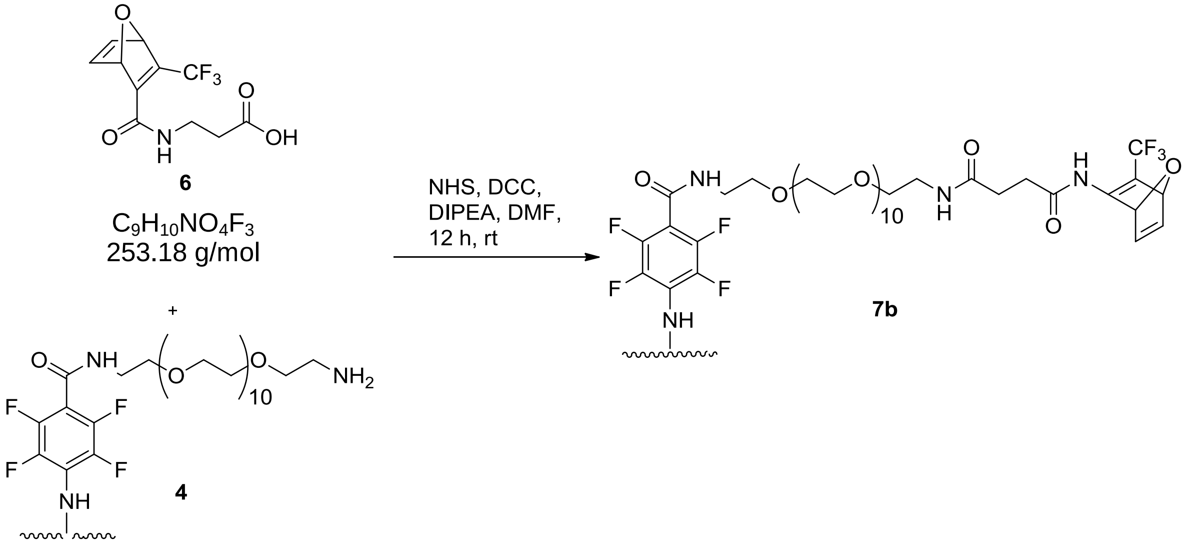

Oxanorbornadiene **6** (16.62 mg, 65.67 µmol, 1.0 equiv), *N*-hydroxysuccinimide (15.12 mg, 131.35 µmol, 2.0 equiv), dicyclohexylcarbodiimide (27.10 mg, 131.35 µmol, 2.0 equiv), and diisopropylethylamine (55.84 µL, 328.37 µmol, 5.0 equiv) were dissolved in dimethylformamide (15 mL) under argon atmosphere. The TPX foils **4** were added and the reaction mixture was stirred at room temperature for 12 h.
Afterwards, the foils were washed with demineralized water, isopropanol and methanol and dried in vacuo.
The modified foils **7b** were not analyzed directly but the reaction between linker **3** and oxanorbornadiene **6** served as a model reaction in order to analyze the best coupling conditions (see chapter 2d below). The amide coupling of the TPX derivative with the approximately 65 PEG units longer linker was performed in the same way as described for **7b**.

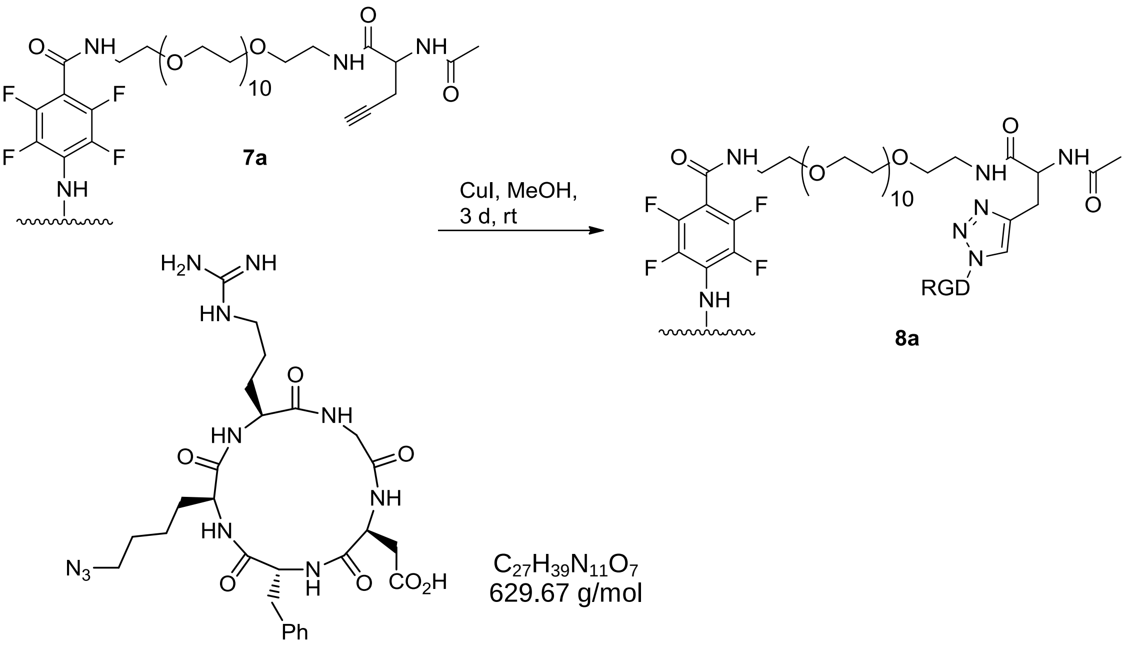

1b

Copper-catalyzed "click" chemistry on the polymer surface was performed by solving *c*RGDfK(N_3) **1b** (3.0 mg, 4.77 µmol) in methanol (20.00 mL) under an argon atmosphere. DIPEA (10.0 µL, 1% in methanol) was added and the reaction mixture was degassed three times, using the "freeze–pump–thraw" degassing method. After adding copper(I) iodide (0.1 mg, 0.48 µmol, 0.10 equiv) and the foils of TPX-derivative **7a**, the mixture was incubated for three days at room temperature. Afterwards, the TPX foils were removed and flushed with a 3% (w/v) solution of Triton X, isopropanol, acetone and methanol and dried in vacuo.

The analysis was carried out as described in the main manuscript. The synthesis of **8c** was performed in the same way as described for **8a**.

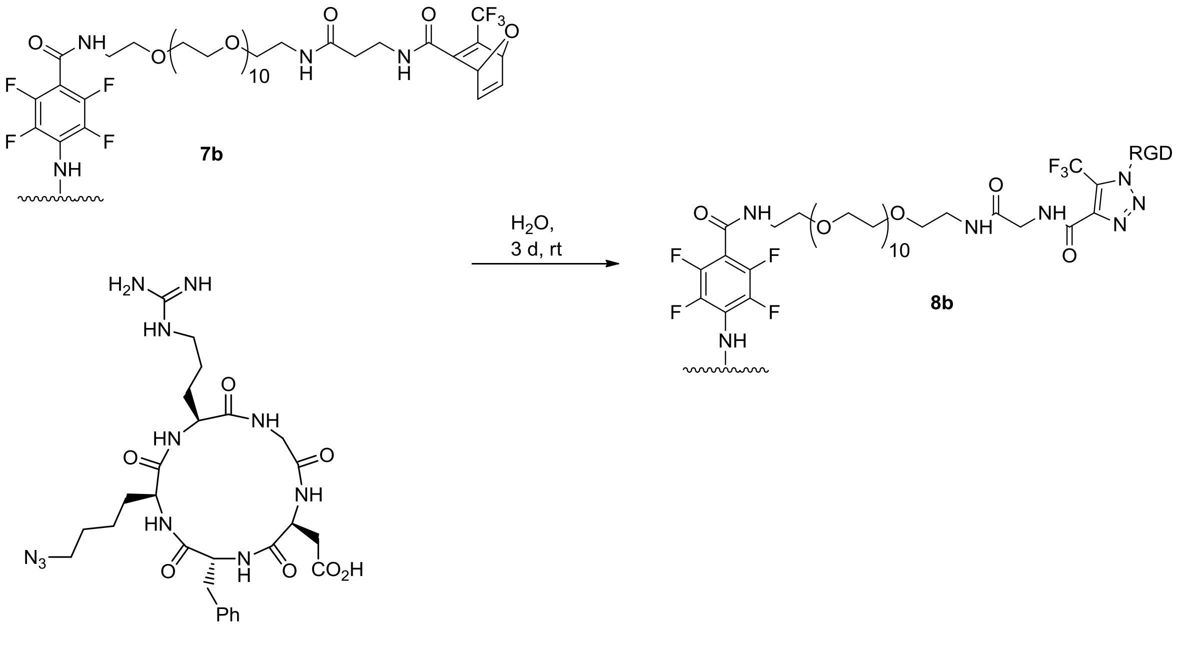

1b

Copper-free "click" chemistry was performed on the TPX-surface by incubating the modified TPX foils **7b** in a solution of cRGDfK(N_3) **1b** (1.5 mg, 2.39 µmol) in water (10.00 mL) for three days at room temperature. Afterwards, the TPX foils were removed and flushed with 3% (w/v) solution of Triton X, isopropanol, acetone and methanol and dried in vacuo.

The analysis was carried out as described in the main manuscript. The synthesis of **8d** was performed in the same way as described for **8b**.

2D. Model reactions

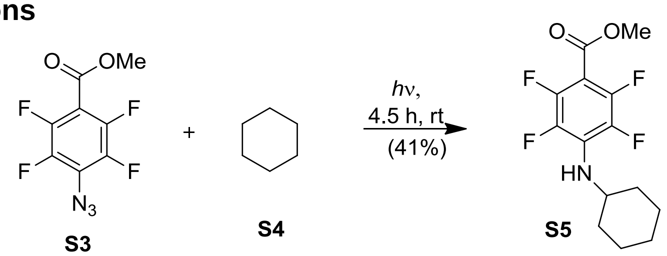

S3 $C_8H_3F_4N_3O_2$ 249.02 g/mol

S4 C_6H_{12} 84.09 g/mol

S5 $C_{14}H_{15}F_4NO_2$ 305.10 g/mol

As a model reaction for the UV-mediated insertion of nitrenes into saturated hydrocarbons, 4-azido-2,3,5,6-tetrafluorobenzoic acid methylester (**S3**, 124.0 mg, 497.95 µmol) was dissolved in cyclohexane (**S4**, 100 mL) and placed into quartz glass flasks under argon atmosphere according to a literature described method of Keana et al (ref. S1). Subsequently, the reaction mixture was exposed for 4.5 hours to a low-pressure mercury lamp.

The excess of cyclohexane was removed in vacuo and the crude product was purified by column chromatography on silica gel (eluation with dichloromethane/hexane 1/3 to dichloromethane/hexane 1/1). 63.5 mg of colorless crystals of 4-cyclohexylamino-2,3,5,6-tetrafluoro-benzoic acid methylester (**S5**, 208 µmol, 41%) were obtained.

Mp 87 °C, TLC: R_F (dichloromethane/hexane 1/2) = 0.18; ^{1}H NMR (400 MHz, $CDCl_3$) δ 1.15–1.29 (m, 3 H, 2 × C*H*H^{ortho} und 1 × C*H*H^{para}), 1.33–1.46 (m, 2 H, 2 × C*H*H^{meta}), 1.63–1.72 (m, 1 H, 1 × CH*H*^{para}), 1.75–1.84 (m, 2 H, 2 × CH*H*^{meta}), 2.02–2.11 (m, 2 H, 2 × CH*H*^{ortho}), 3.63–3.74 (m, 1 H, C*H*), 3.93 (s, 3 H, C*H$_3$*), 4.02–4.11 (m, 1H, N*H*) ppm; ^{13}C NMR (100 MHz, $CDCl_3$) δ 24.84 (*C*H$_2^{ortho}$, *C*H$_2^{meta}$), 34.66 (*C*H$_2^{para}$), 53.58 (*C*H$_3$), 53.67 (*C*H), 130.72, 135.31, 137.78, 144.06, 145.61, 148.11, (*C*H$^{arom.}$), 161.44 (*C*O$_2$Me) ppm; HRMS: $C_{14}H_{15}F_4NO_2Na$ $[M + Na]^+$ calcd for 328.0937, found: 328.0944

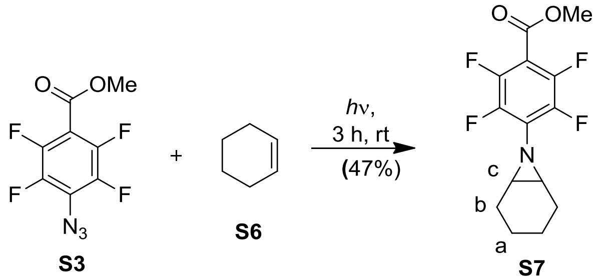

S3 $C_8H_3F_4N_3O_2$ 249.02 g/mol

S6 C_6H_{10} 82.08 g/mol

S7 $C_{14}H_{13}F_4NO_2$ 303.09 g/mol

As a model reaction for the insertion of azides into unsaturated systems, 4-azido-2,3,5,6-tetrafluorobenzoic acid methylester (**S3**, 62.0 mg, 248.98 µmol) was dissolved in cyclohexene (**S6**, 50 mL) under an argon atmosphere. This solution was stirred in a quartz glass flask and simultaneously exposed for 3 hours to a low-pressure mercury lamp. Excess of cyclohexene was removed in vacuo and the crude product was purified by column chromatography on silica gel (dichloromethane/petroleum ether 1/3). 35.8 mg of colorless crystals of 4-(7-aza-bicyclo[4.1.0]hept-7-yl)-2,3,5,6-tetrafluorobenzoic acid methylester (**S7**, 118.12 µmol, 47%) were obtained.

Mp 60–65 °C, TLC: R_F (dichloromethane/hexane 1/3) = 0.17; ^{1}H NMR (400 MHz, $CDCl_3$) δ 1.26–1.39 (m, 2 H, 2 × C*H*H^a), 1.45–1.58 (m, 2 H, 2 × CH*H*^{a}), 1.86–1.97 (m, 2 H, 2 × C*H*H^b), 2.06–2.18 (m, 2 H, 2 × CH*H*^{b}), 2.56–2.61 (m, 2 H, C*H$_2$*^{c}), 3.92 (s, 2 H, C*H$_3$*) ppm; ^{13}C NMR (100 MHz, $CDCl_3$) δ 20.01 (*C*H$_2^a$), 23.73 (*C*H$_2^b$), 41.63 (*C*H^c), 52.96 (*C*H$_3$), 103.85 (*C*$^{arom.}$CO), 127.58 (*C*$^{arom.}$N$_3$), 139.94, 142.55, 144.58, 147.12 (*C*$^{arom.}$F), 160.98 (*C*O$_2$Me) ppm; HRMS: $C_{14}H_{13}F_4NO_2Na$ $[M + Na]^+$ calcd for 326.0780, found: 326.0775.

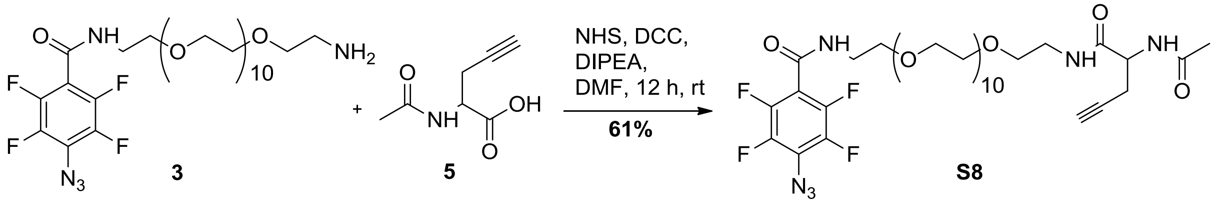

$C_{31}H_{51}F_4N_5O_{12}$ 761.35 g/mol

$C_7H_9NO_3$ 155.06 g/mol

$C_{38}H_{58}F_4N_6O_{14}$ 898.39 g/mol

Linker **3** (20.0 mg, 26.27 µmol, 1.0 equiv) and 2-acetamino-pent-4-ynoic acid (**5**, 4.10 mg, 26.27 µmol, 1.0 equiv) were dissolved in dimethylformamide (1 mL) under an argon atmosphere. N-hydroxysuccinimide (6.0 mg, 52.54 µmol, 2.0 equiv), dicyclocarbodiimide (10.8 mg, 52.54 µmol, 2.0 equiv) and N,N-diisopropylethylamine (22.3 mL, 131.35 µmol, 5.0 equiv) were added and the reaction mixture was stirred for 12 hours at room temperature. The solvent was removed in vacuo and the crude product was purified by column chromatography on silica gel (dichloromethane/methanol 95/5). Product **S8** (14.5 mg, 16.14 µmol, 61%) was obtained as a colorless oil.
TLC: R_F (dichloromethane/methanol 95/5) = 0.24; 1H NMR (400 MHz, $CDCl_3$) δ 2.03 (s, 3 H, $CH_3{}^{acetyl}$), 2.14 (t, $J_{H,H}$ = 2.56 Hz, 1 H, CH ^{alkyne}), 2.60 (ddd, $J_{H,H}$ = 16.04 Hz, $J_{H,H}$ = 5.46 Hz, $J_{H,H}$ = 2.39 Hz, 1 H CH^{β}), 2.75 (ddd, 3 $J_{H,H}$ = 16.04 Hz, $J_{H,H}$ = 3.75 Hz, $J_{H,H}$ = 2.39 Hz, 1 H CH^{β}), 3.39–3.85 (m, 48 H, $CH_2{}^{PEG}$), 4.50–4.56 (m, 1 H, CH^{α}), 6.61 (d, $_{JH,H}$ = 7.51 Hz, 1 H, NHAc), 6.94–7.01 (m, 1 H, NH^{PEG}), 7.14–7.21 (m, 1 H, NH^{PEG}) ppm; ^{13}C NMR (100 MHz, $CDCl_3$) δ 22.75 (CH_3), 23.30 ($CH_2{}^{\beta}$), 39.68, 40.21 ($CH_2{}^{PEG}$), 51.69 ($CH_2{}^{\alpha}$), 69.58–71.84 ($CH_2{}^{PEG}$), 112.19 (C $^{arom.CO}$), 121.57 (C $^{arom.N}$3), 139.32, 142.65, 142.94, 145.31 (C $^{arom.F}$), 157.95 ($CONH^{PEG}$), 169.92 ($CONH^{AA}$) ppm; HRMS: $C_{38}H_{58}F_4N_6O_{14}Na$ [M+Na]+ calc: 921.3845, found: 921.3840.

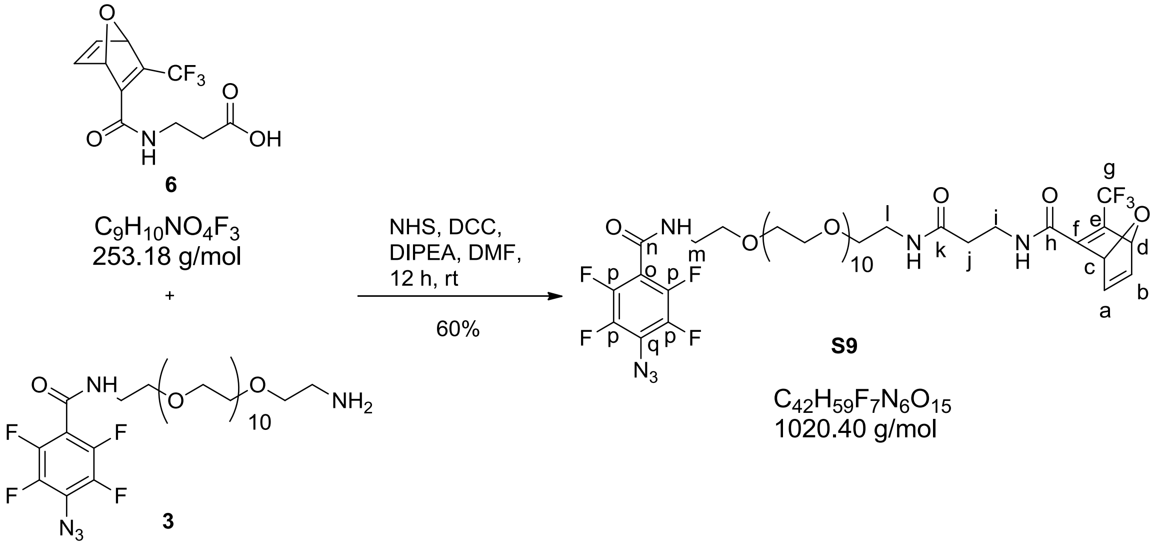

$C_9H_{10}NO_4F_3$ 253.18 g/mol

$C_{42}H_{59}F_7N_6O_{15}$ 1020.40 g/mol

$C_{31}H_{51}F_4N_5O_{12}$ 761.35 g/mol

Oxanorbornadiene derivative **6** (14.6 mg, 52.54 µmol, 1.0 equiv) was added to a solution of linker **3** (40.0 mg, 52.54 µmol, 1.0 equiv) in dimethylformamide (2.0 mL). Subsequently, *N*-hydroxysuccinimide (12.1 mg, 105.08 µmol, 2.0 equiv), dicyclocarbodiimide (21.7 mg, 105.08 µmol, 2.0 equiv), and *N,N*-diisopropylethylamine (43.4 µL, 262.69 µmol, 5.0 equiv) were added and the reaction mixture was stirred for 12 hours at room temperature. The precipitate was filtered off and the filtrate was dried in vacuo. The crude product was purified by column chromatography on Sephadex (methanol) yielding **S9** (32.1 mg, 31.26 µmol; 60%) as colorless crystals.
Mp decomposition >140 °C TLC: R_F (dichloromethane/methanol 9.5/1) = 0.5; ^{1}H NMR (400 MHz, $CDCl_3$) δ 2.48 (t, 2 H, $J_{H,H}$ = 5.97 Hz, H^j), 3.39–3.69 (m, 50 H, PEG and H^i), 6.78 (s^{br}, 1 H, N*H*), 7.19 (s^{br}, 1 H, N*H*), 7.85–7.80 (m, 4 H, $H^{a\text{-}d}$) ppm; ^{13}C NMR (100 MHz, $CDCl_3$) δ 35.08 (C^j), 35.98 (C^i),

39.78, 40.63 ($C^{l,m}$), 69.78–70.88 (C^{PEG}), 101,.84 (C^{o}), 116.77 (quart, $J_{C,F}$ = 37.2 Hz, C^{e}), 118.62 (quart, $J_{C,F}$ = 19.6 Hz, C^{f}), 120.66–120.70 (C^{q}), 120.81 (quart, $J_{C,F}$ = 122.4 Hz, C^{g}), 126.29, 134.49 ($C^{c,d}$), 140.50, 142.94, 143.14, 145.51 (m, C^{p}), 144.79 (quart, $J_{C,F}$ = 6.6 Hz, C^{b}) 147.21 (C^{a}), 157.57, 160.82 ($C^{h,n}$), 172.21 (C^{k}) ppm; HRMS: $C_{42}H_{60}F_7N_6O_{15}$ $[M + H]^+$ calcd for 1021.4005, found: 1021.3970, $C_{42}H_{59}F_7N_6O_{15}Na$ $[M + Na]^+$ calcd for 1043.3824, found: 1043.3837.

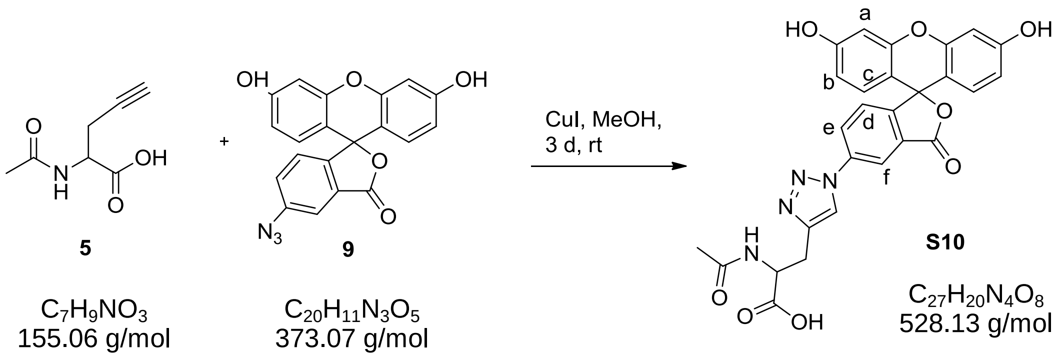

5-Azidofluorescein **9** (4.16 mg, 26.8 µmol, 1.0 equiv) and 2-acetylamino-pent-4-ynoic acid (**5**, 10 mg, 26.8 µmol, 1.0 equiv) were dissolved in methanol (1 mL) under argon atmosphere. DIPEA (10.0 µL, 1% in methanol) was added and the reaction mixture was degassed three times using the “freeze–pump–thraw” degassing method. After adding copper(I) iodide (0.5 mg, 2.68 mmol, 0.1 equiv), the mixture was stirred for three days at room temperature. The solvent was removed in vacuo and the crude product was purified by column chromatography on silica gel (dichloromethane/methanol 93/7 → 85/15 → 75/25 → 60/40) to yield the “click“ product **S10** (10.2 mg, 19.32 µmol; 72%) as a red oil.
TLC: R_F (dichloromethane/methanol 9.5/1) = 0.45; ^{1}H NMR (400 MHz, D_2O) δ 2.00 (s, 3 H CH_3^{Acetyl}), 3.10–3.48 (m, 2 H, $CH^{β}$), 4.58–4.60 (m, 1 H, $CH^{α}$), 6.62–6.79 (m, 4 H, CH^{b} CH^{c}), 7.21–7.34 (m, 2 H, CH^{a}), 7.48-7.59 (m, 1 H, CH^{f}), 7.94–8.06 (m, 1 H CH^{e}), 8.16–8.28 (m, 1 H, CH^{d}), 8.44 (s, 1 H, $CH^{Triazol.}$) ppm, traces of copper inhibited the recording of a ^{13}C NMR spectra.

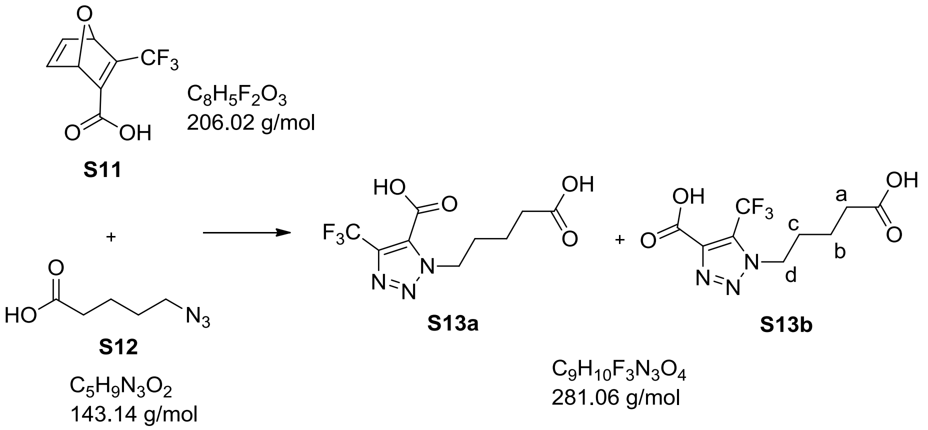

As a model reaction for the copper-free cycloaddition, building block **S11** (40 mg, 194.16 µmol, 1.0 equiv) was mixed with 5-azido-pentanoic acid (**S12**, 27.8 mg, 194.16 µmol, 1.0 equiv) in methanol (3 mL) and stirred for 12 hours at ambient temperature. The solvent was evaporated and the two diastereomers were separated using preparative HPLC (C18-$P_{[A]}$) (H_2O 95:5 {5 min}, 95:5→9:10 {5 min}, 90:10→70:30 {20 min}, 70:30→50:50 {20 min}, 50:50→0:100 {10 min}, 4 mL/min → 5 mL/min). Isomer S13a was obtained after t_R= 42–44 min in 22% yield (12.1 mg, 43.05 µmol) and isomer S13b after t_R 55–56 min in 32% yield (12.1 mg, 43.05 µmol). Both isomers were collected as colorless oils.
HR-MS: $C_9H_9F_3N_3O_4$ $[M-H]^-$ calc: 280.0545, found: 280.0541.
S13a: ^{1}H NMR (400 MHz, MeOD) δ 1.69 (quint, $J_{H,H}$ = 7.54 Hz, 2 H, H^{b}), 2.03 (quint, $J_{H,H}$ = 7.56 Hz, 2 H, H^{c}), 2.40 (t, $J_{H,H}$ = 7.21 Hz, 2 H, H^{a}), 4.83 (t, $J_{H,H}$ = 7.53 Hz, 2 H, H^{d}) ppm; ^{13}C NMR (125 MHz, MeOD) δ 22.78 (C^{b}), 30.51 (C^{c}), 33.98 (C^{a}), 51.80 (C^{d}), 121.62 ($J_{C,F}$ = 268.03 Hz, CF_3), 129.88 (C=CCF_3), 140.04 (quart, $J_{C,F}$ = 39.84 Hz, CCF_3), 159.29 ($CO_2H^{Triazole}$), 176.89 (CO_2H-C^{a}) ppm; ^{19}F NMR (565 MHz, MeOD) δ = -60.6 ppm.

S13b: ^{1}H NMR (400 MHz, MeOD) δ 1.70 (quint, $J_{H,H}$ = 7.48 Hz, 2 H, H^{b}), 2.03 (quint, $J_{H,H}$ = 7.62 Hz, 2 H, H^{c}), 2.41 (t, $J_{H,H}$ = 7.18 Hz, 2 H, H^{a}), 4.70 (t, $J_{H,H}$ = 7.65 Hz, 2 H, H^{d}) ppm; ^{13}C NMR (100 MHz, MeOD) δ 22.73 (C^{b}), 30.45 (C^{c}), 33.87 (C^{a}), 52.61 (quart, $J_{C,F}$ = 2.73 Hz ,C^{d}), 120.86 (quart, $J_{C,F}$ = 269.69 Hz, CF_3), 129.52 (quart, $J_{C,F}$ = 42.27 Hz, CCF_3), 140.96 (C=CCF_3), 161.62 ($CO_2H^{Triazole}$), 176.68 (CO_2H-C^{a}) ppm; ^{19}F NMR (565 MHz, MeOD) δ 56.6 ppm.

3. Analysis of the nitrene insertion with ATR-FT-IR

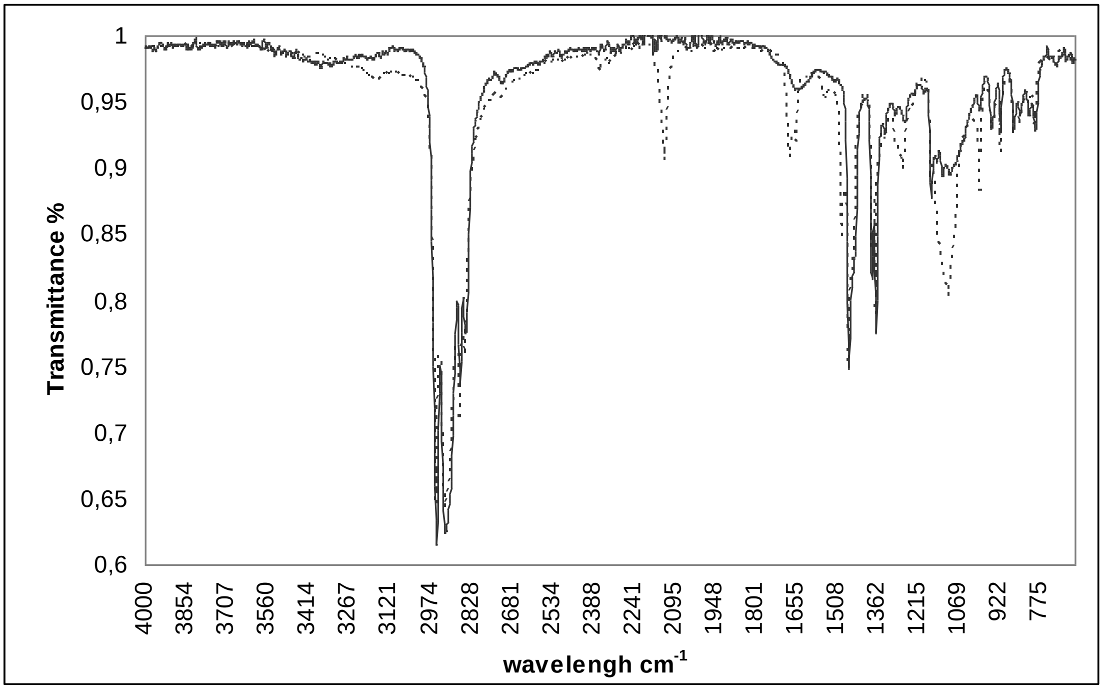

Figure S1: ATR-FT-IR spectra of TPX dip coated in a solution of linker **3** before (dashed line) and after UV irradiation and exhaustive washing with demineralized water, isopropanol and methanol (continuous line).

4. High-resolution XPS spectra of untreated TPX 2 and plasma-treated as well as modified TPX 4

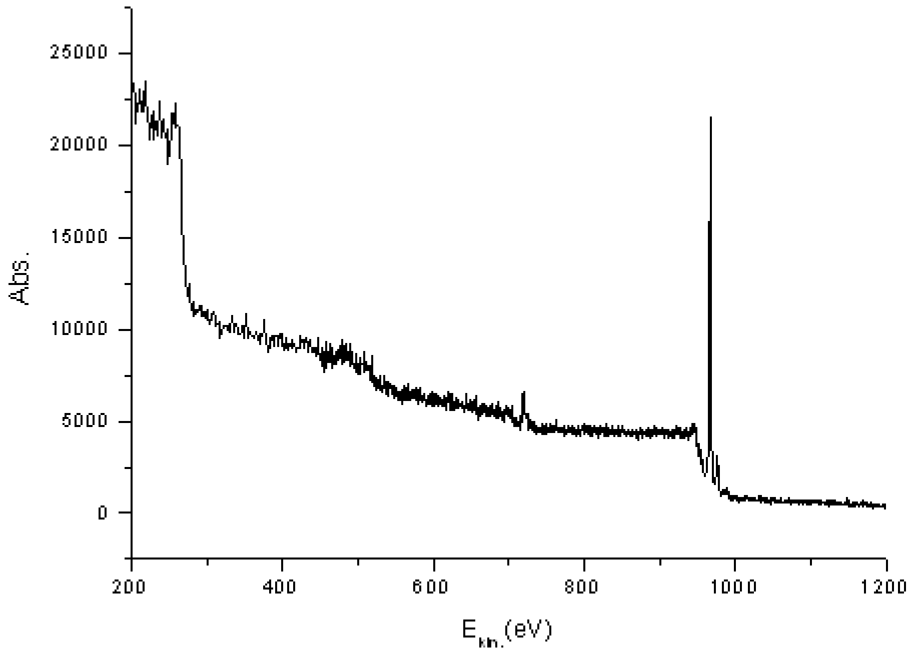

Figure S2: TPX without any treatment (pass 100).

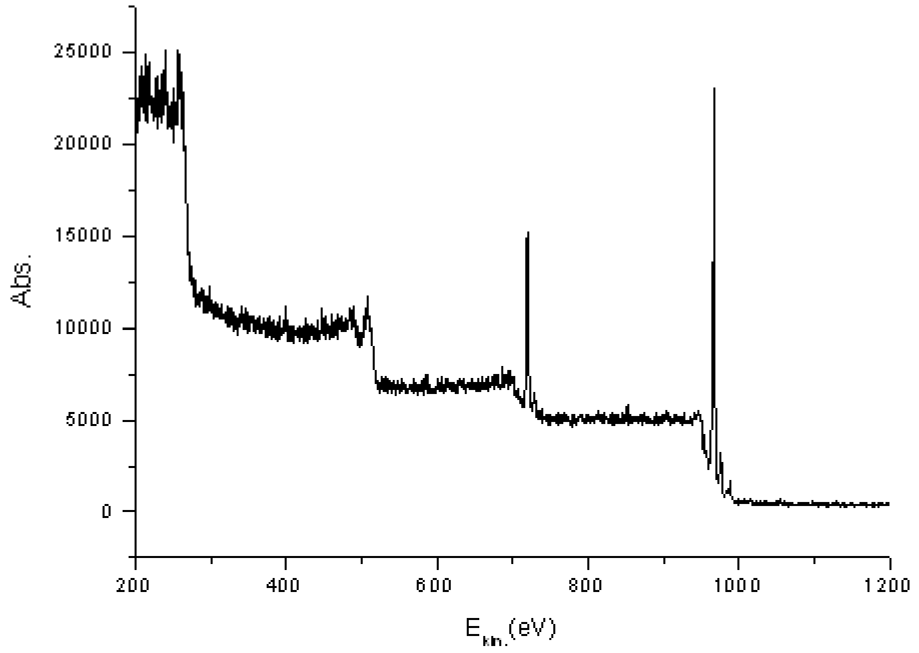

Figure S3: TPX plasma-treated (pass 100).

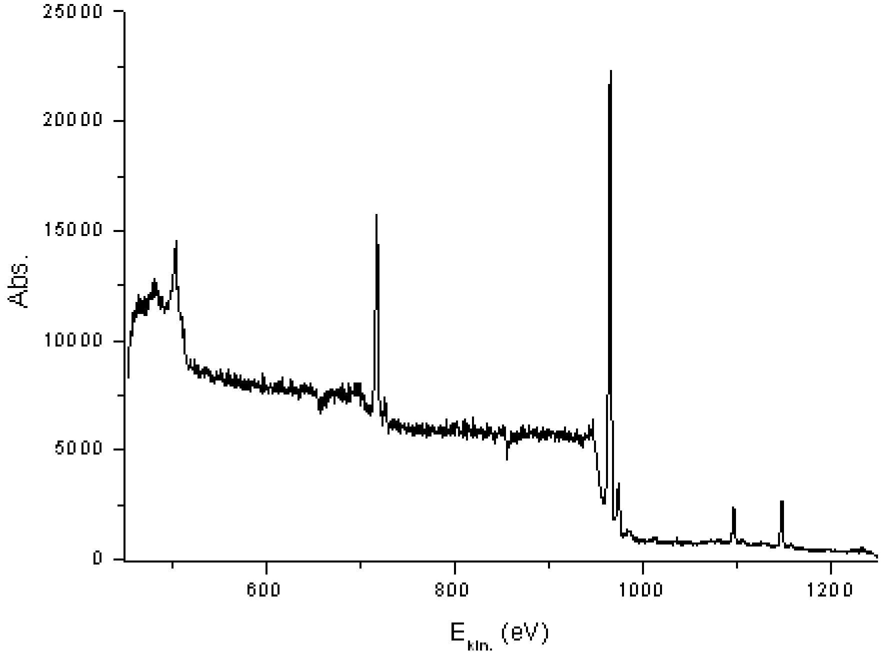

Figure S4: TPX derivative **4** without plasma treatment before the dip-coating process (pass:150).

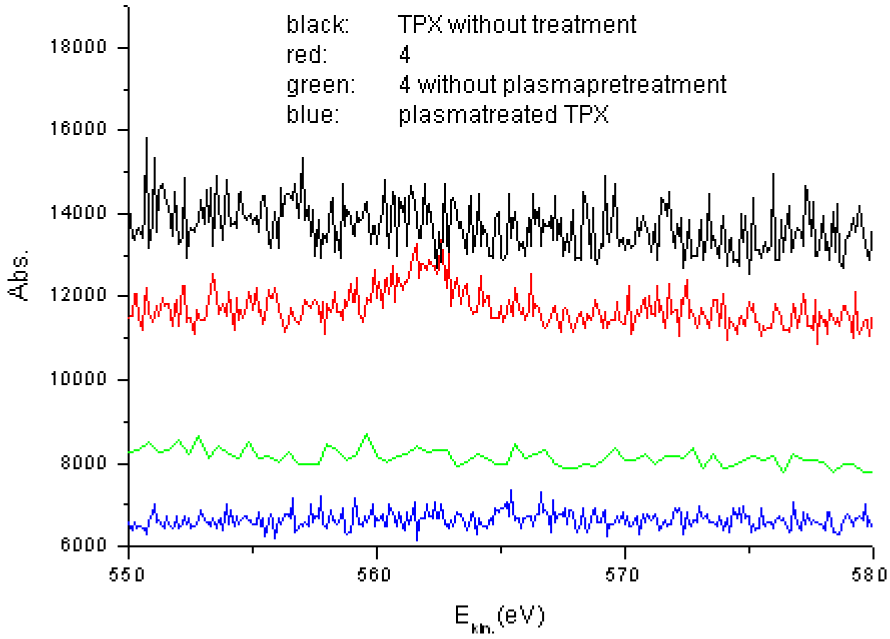

Figure S5: Comparison of the high-resolution fluorine peak of different TPX derivatives (black, red green: Pass 150, blue Pass: 100).

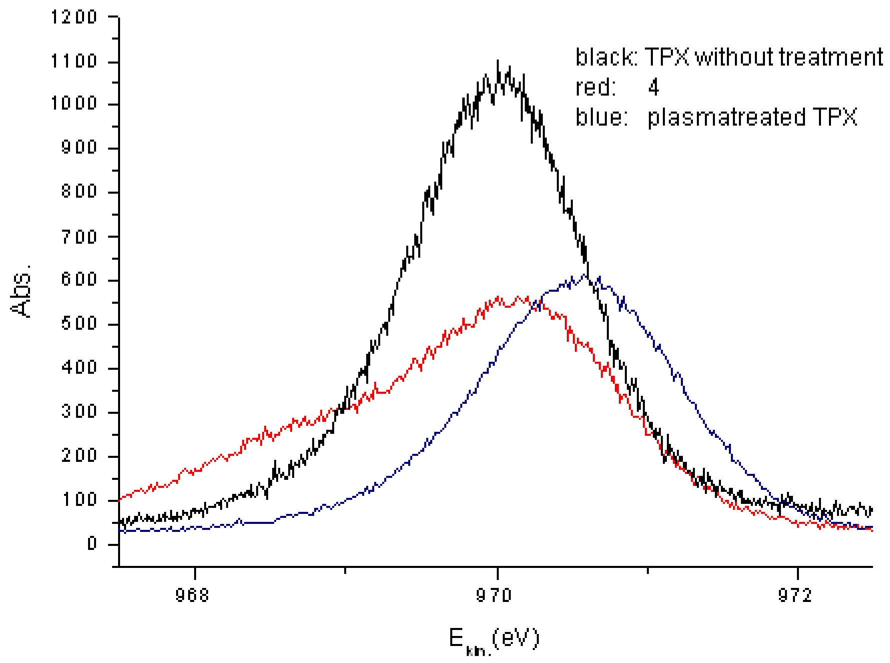

Figure S6: Comparison of the high-resolution carbon peak of different TPX derivatives (Pass: 20).

5. Quantification of the linker on the polymer surface

The area under the Gauss fits of the peaks was determined and compared to the integral under the Gauss fits of the carbon peak. The latter was used as standard because the polymer backbone mainly consists of carbon (hydrogen cannot be measured by XPS).

Plasma treatment led to an increase of oxygen and nitrogen on the surface. The percentage was 1:55 while no nitrogen could be detected on the surface of the nonfunctionalized polymer. Insertion of the linker further raised the ratio to 1:35. The ratio of fluorine to carbon was 1:60. Oxygen was already present on the surface of untreated TPX (1:18). Plasma treatment led to a ratio of 1:5 and linker insertion to a ratio of 1:3.5.

6. UV spectra of TPX derivatives with fluorescein

6A. Fluorescein coupled by Cu-catalyzed "click" chemistry

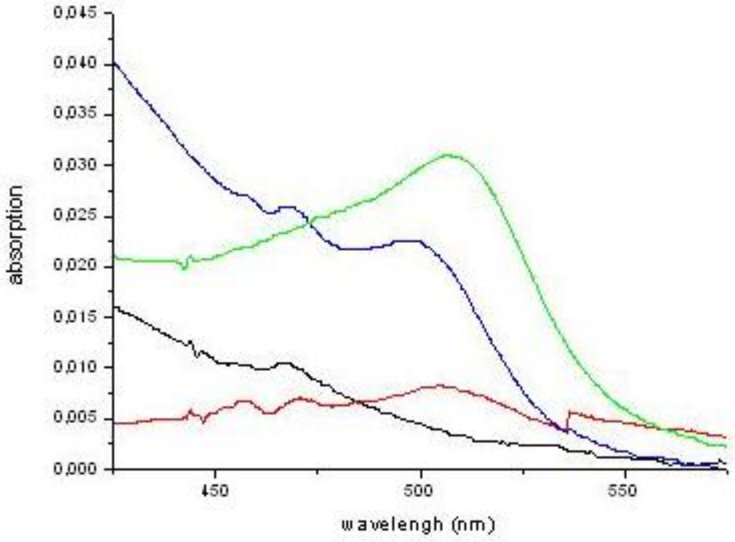

Figure S7: Blue: Fluorescein-functionalized TPX **8c**; green: Fluorescein-functionalized TPX **8c** with elongated linker; black: Alkyne functionalized TPX **7a**, starting material for "click" chemistry; red: Negative control for the click reaction: unfunctionalized TPX was treated the same way as **7a** in order to exclude adhesion of the dye.

6B. Fluorescein coupled by Cu-free "click" chemistry

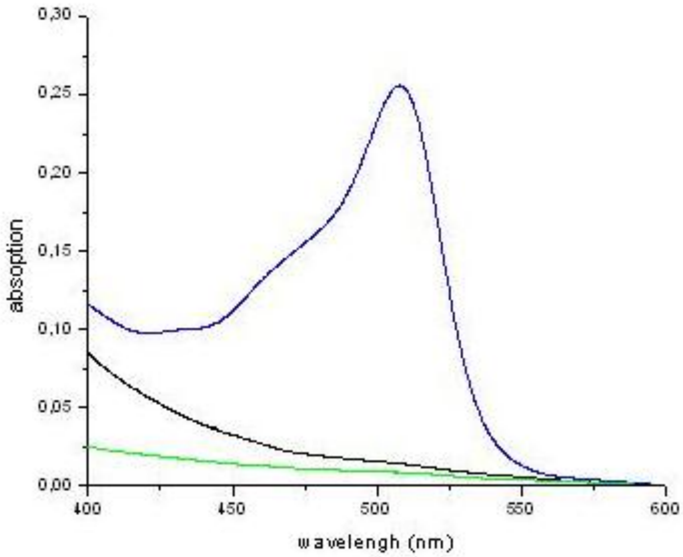

Figure S8: Blue: Fluorescein-functionalized TPX **8d**; green: Fluorescein-functionalized TPX **8d** with elongated linker; black: Alkyne functionalized TPX **7b**, starting material for "click" chemistry.

7. Contact angles

Table S1:

No.	Contact angle	distribution
**2**	107°	1.2°
**2** after plasma-treatment after 1 day	46.1°	6.5°
**2** after plasma-treatment after 1 week	69.4°	5.2°
**2** after plasma-treatment after 2 months	74.7°	1.7°
**4**	74.7°	4.1°
**7a**	97.9	6.4
**7b**	72.8°	5.8°
**8a**	92.8°	3.1°
**8b**	77.7°	4.2°

8. Measurements of carbon dioxide and oxygen permeability through TPX and functionalized TPX membranes

	thickness of TPX membrane		O_2 permeability according to ISO 15105-2
	µm (min)	µm (max)	[$cm^3\ m^{-2}\ d^{-1}\ bar^{-1}$]
Unmodified membrane	53.4	54.4	22500
	53.7	56.8	24600
Modified membrane	54.3	55.6	21900
	52.0	52.7	20400

9. NMR spectra

9.1. Aryl azide S2a

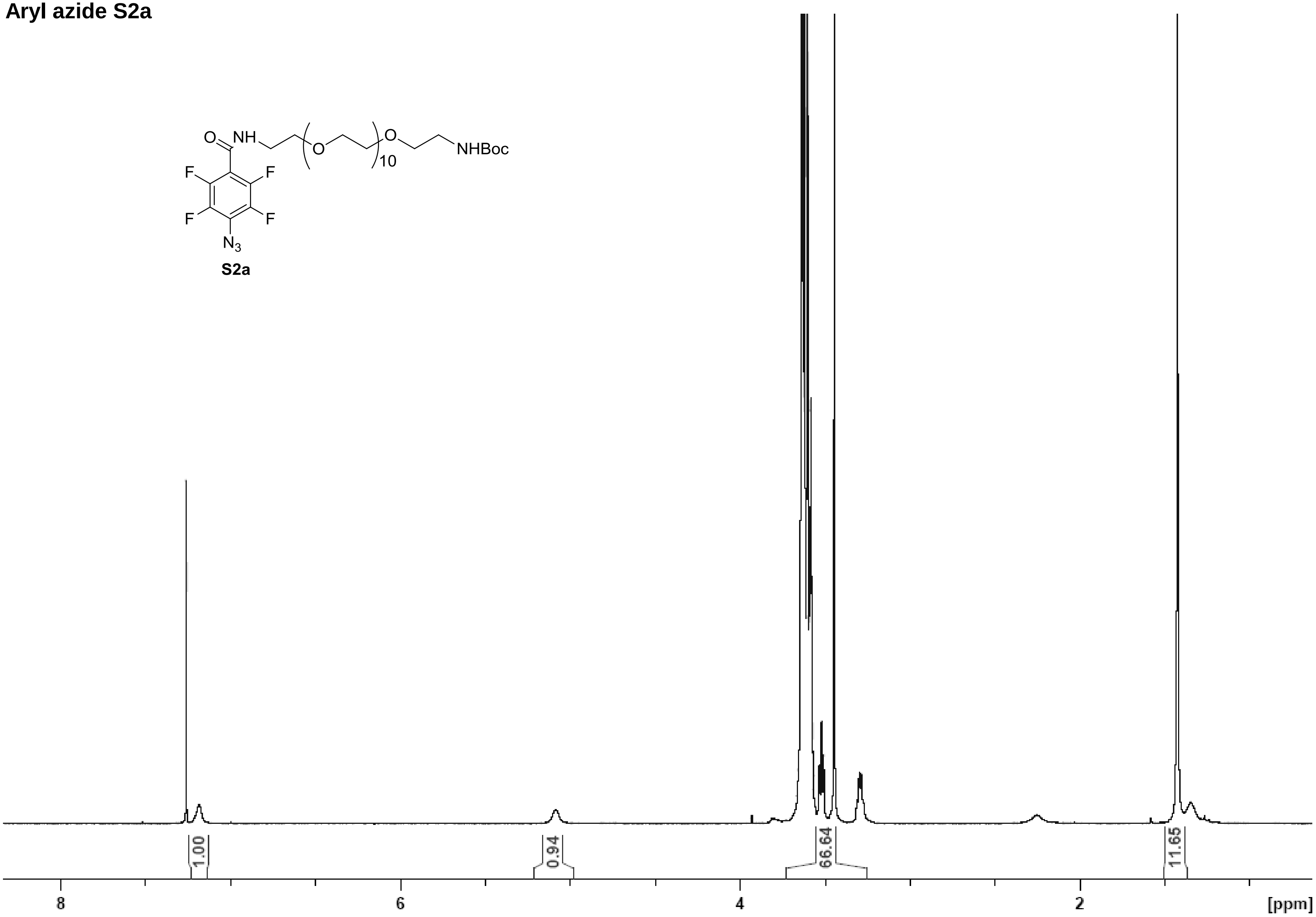

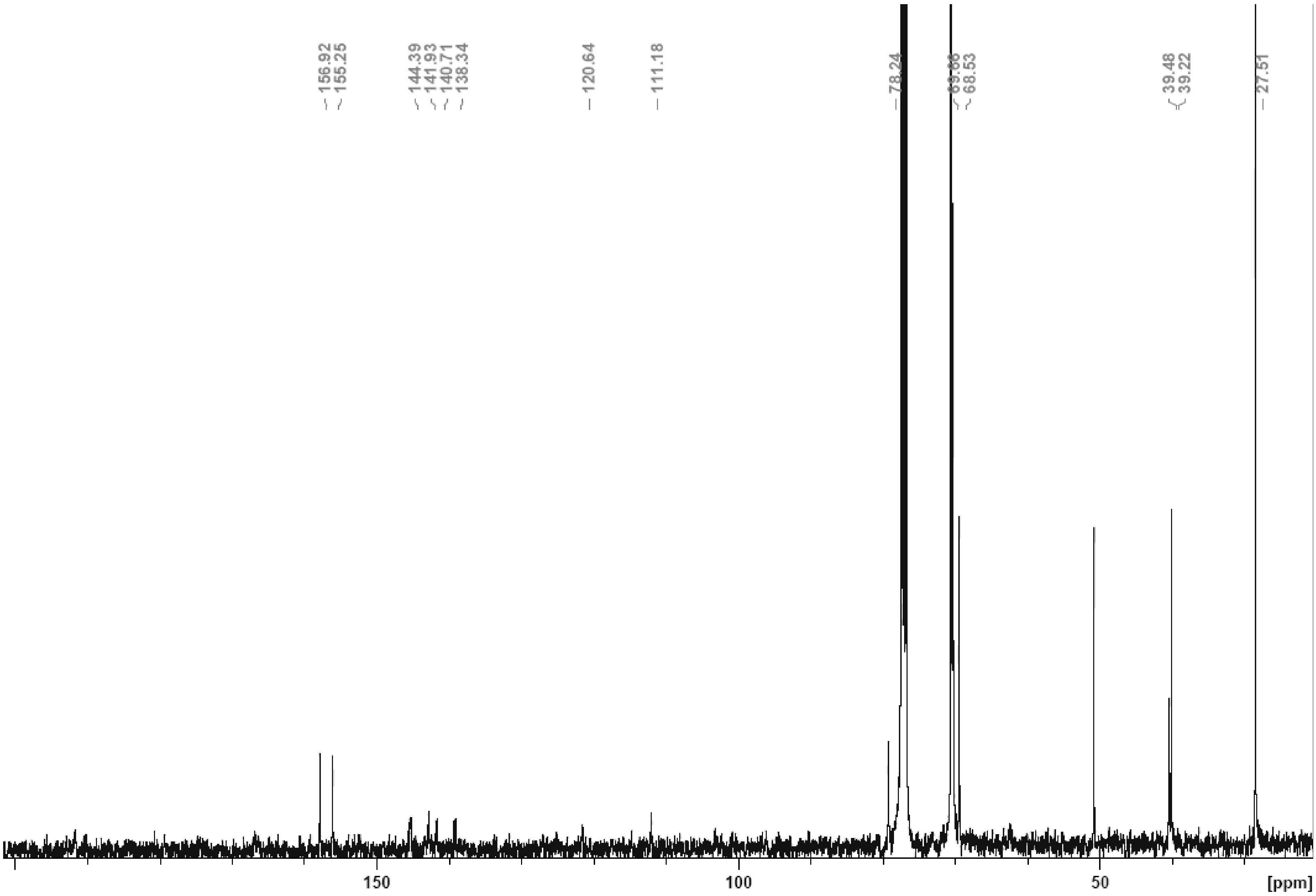

156.92
155.25
144.39
141.93
140.71
138.34
120.64
111.18
78.24
69.66
68.53
39.48
39.22
27.51
150
100
50
[ppm]

9.2. Aryl azide 3

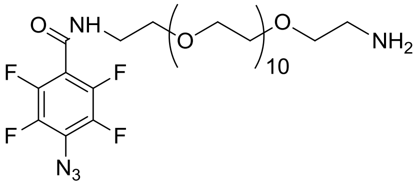

3

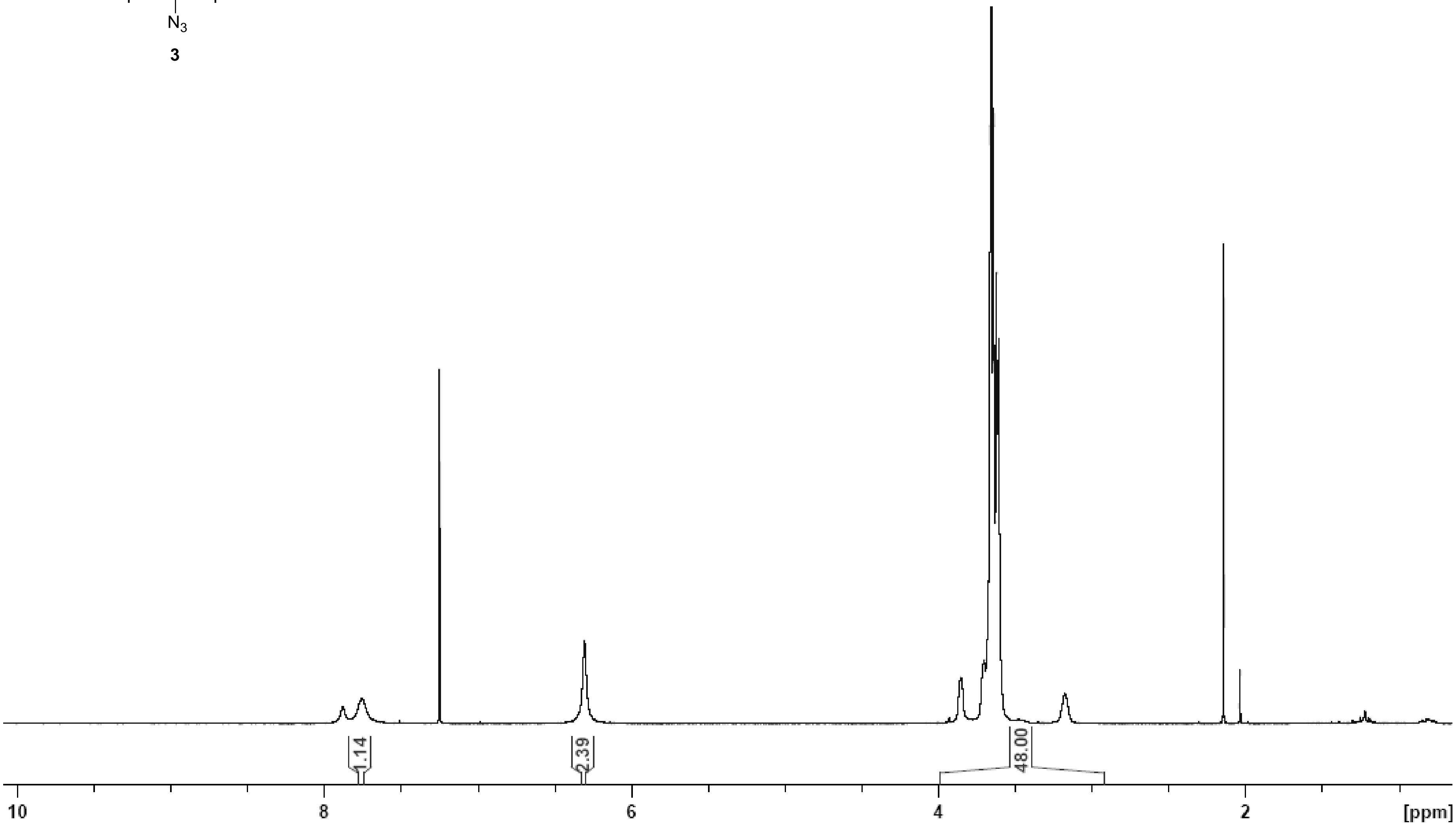

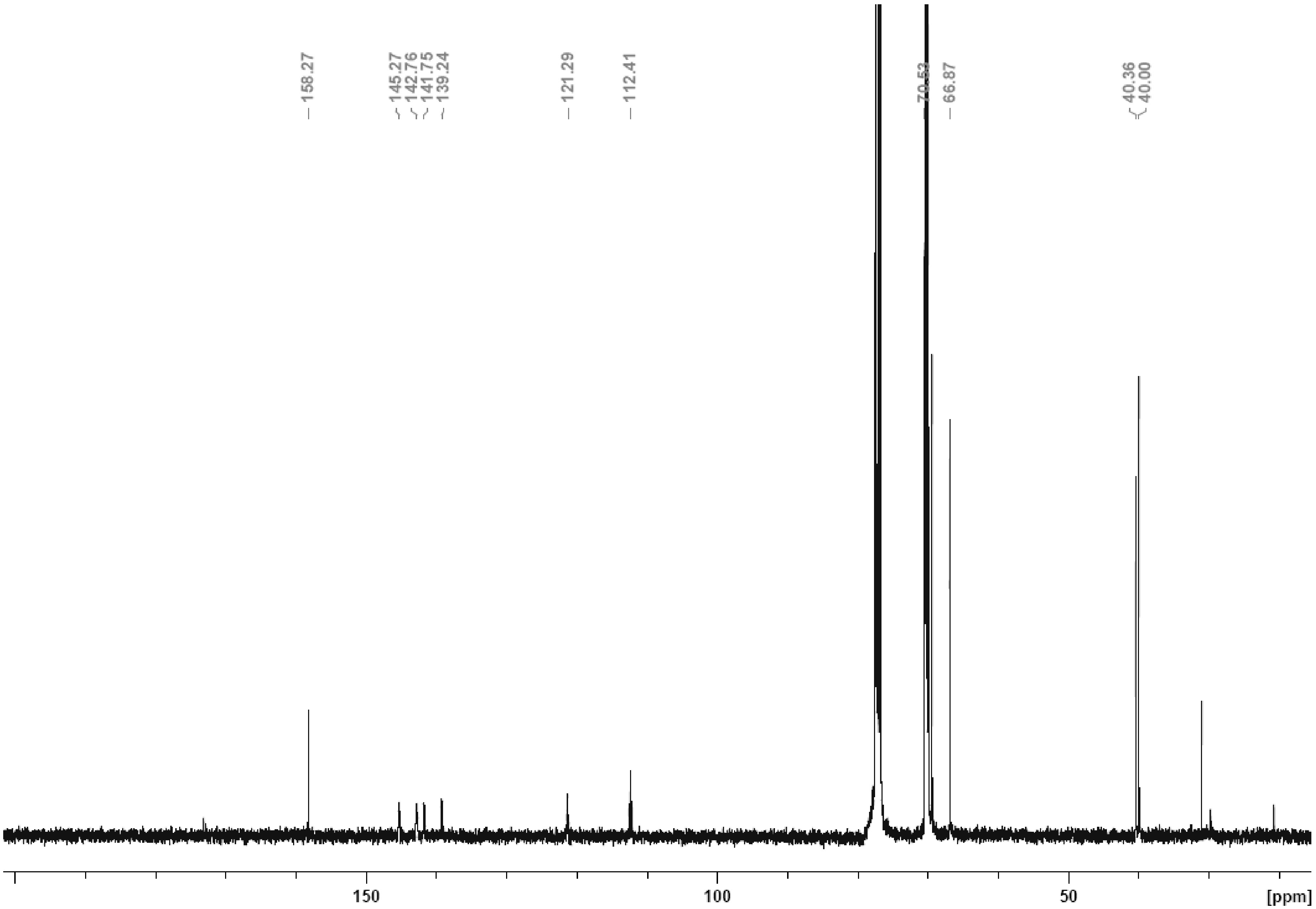
158.27
145.27
142.76
141.75
139.24
121.29
112.41
70.53
66.87
40.36
40.00
150
100
50
[ppm]

9.4. Aryl aziridine S7

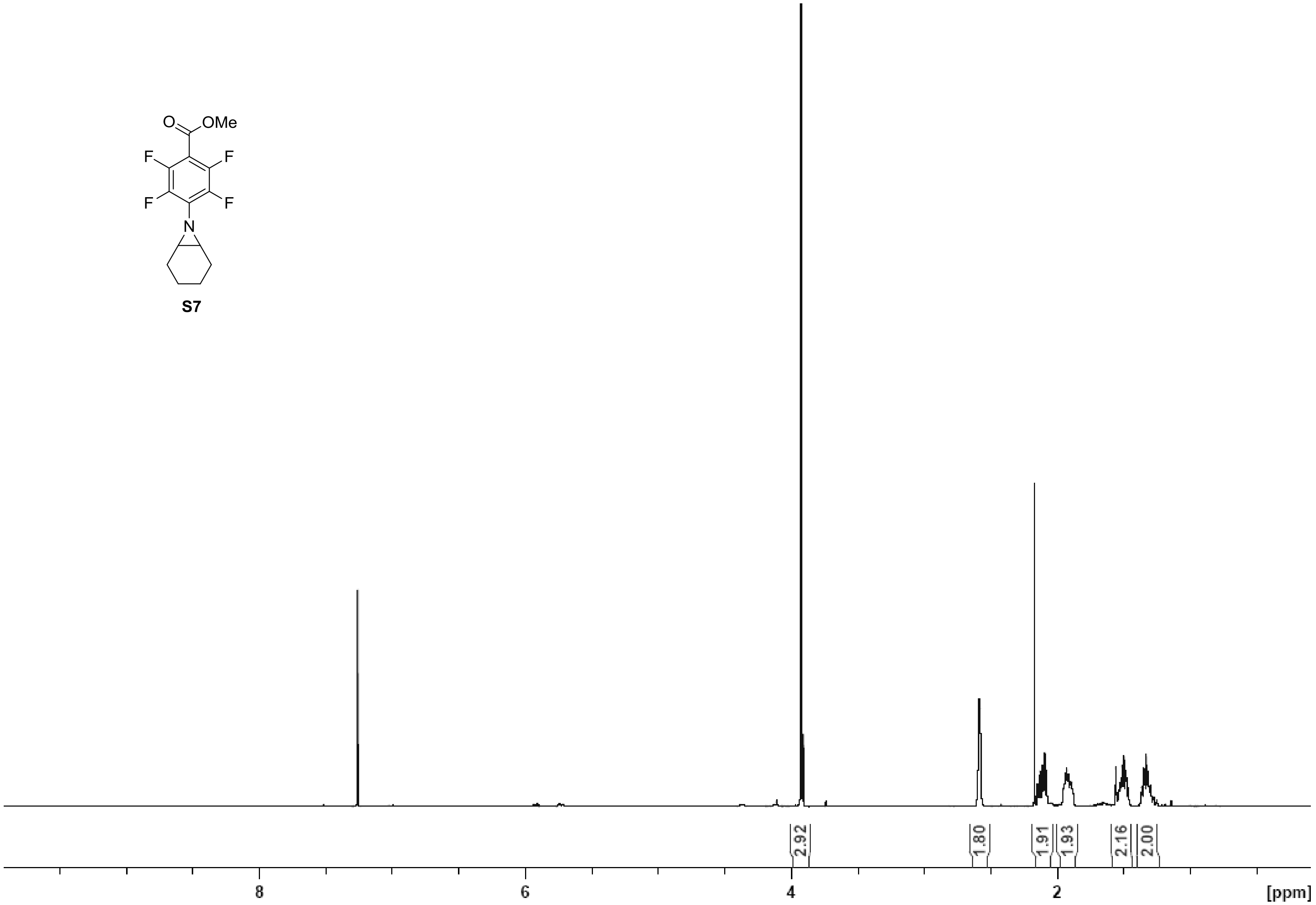

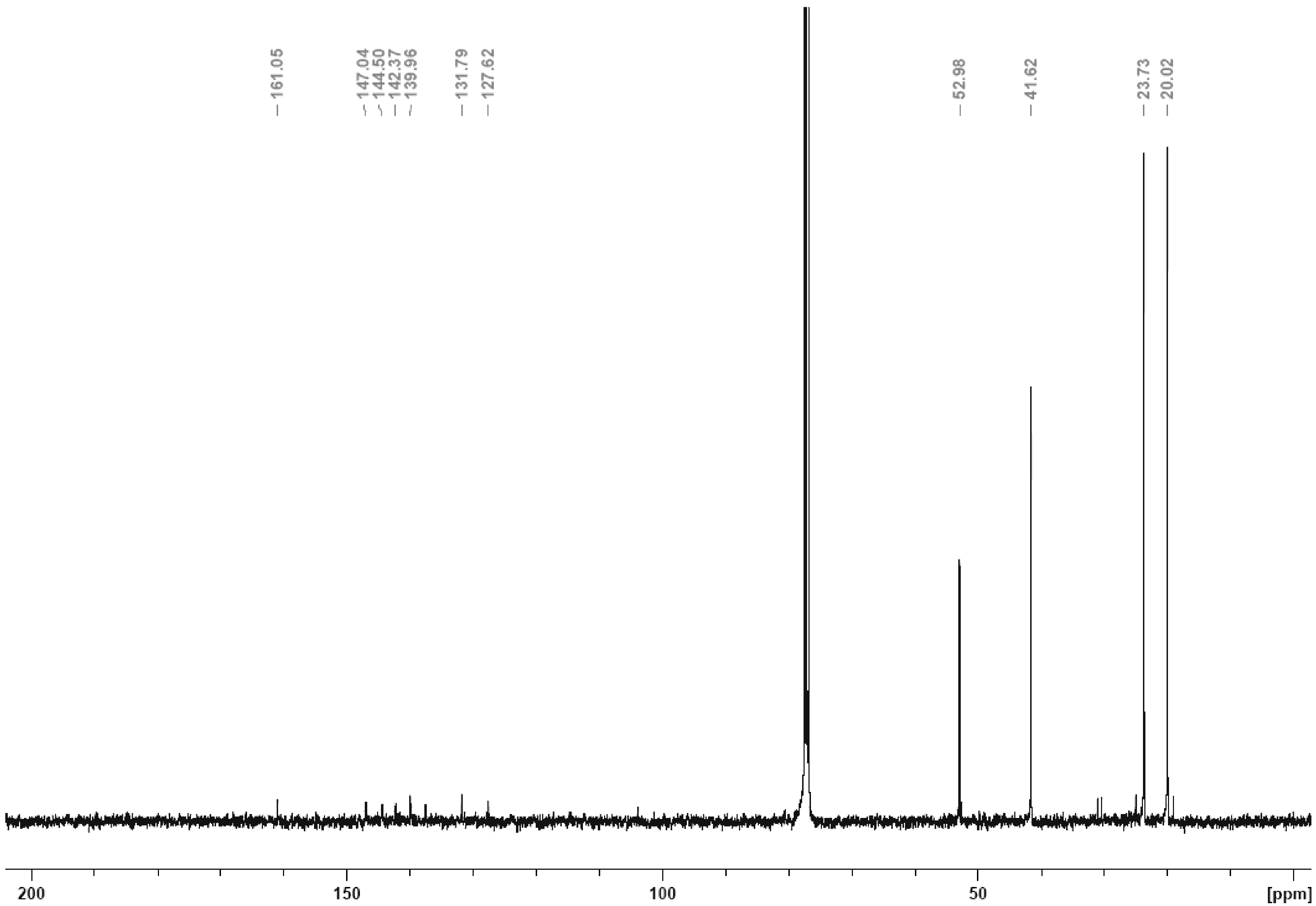
161.05
147.04
144.50
142.37
139.96
131.79
127.62
52.98
41.62
23.73
20.02
200
150
100
50
[ppm]

9.5. Aryl azide S8

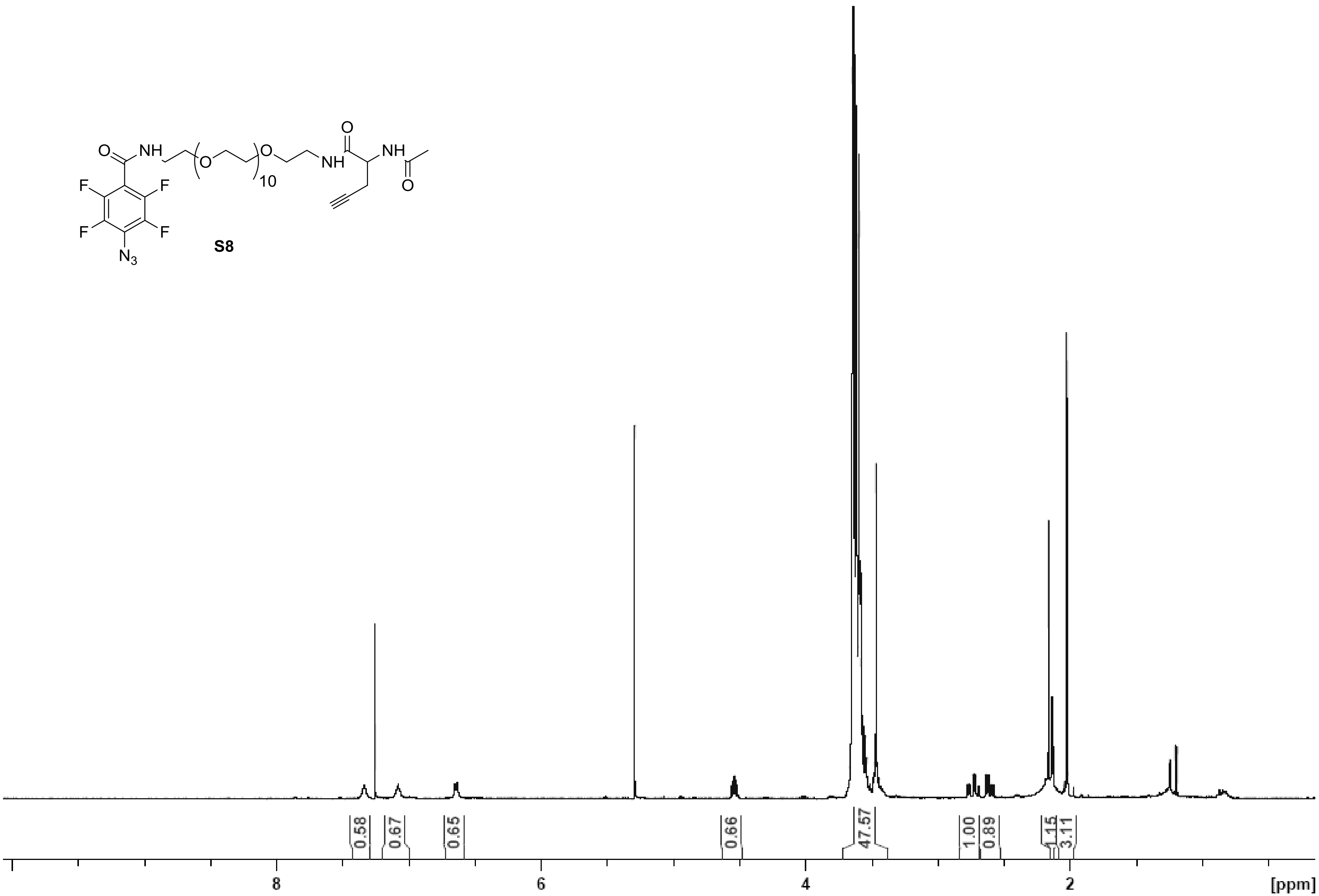

S8

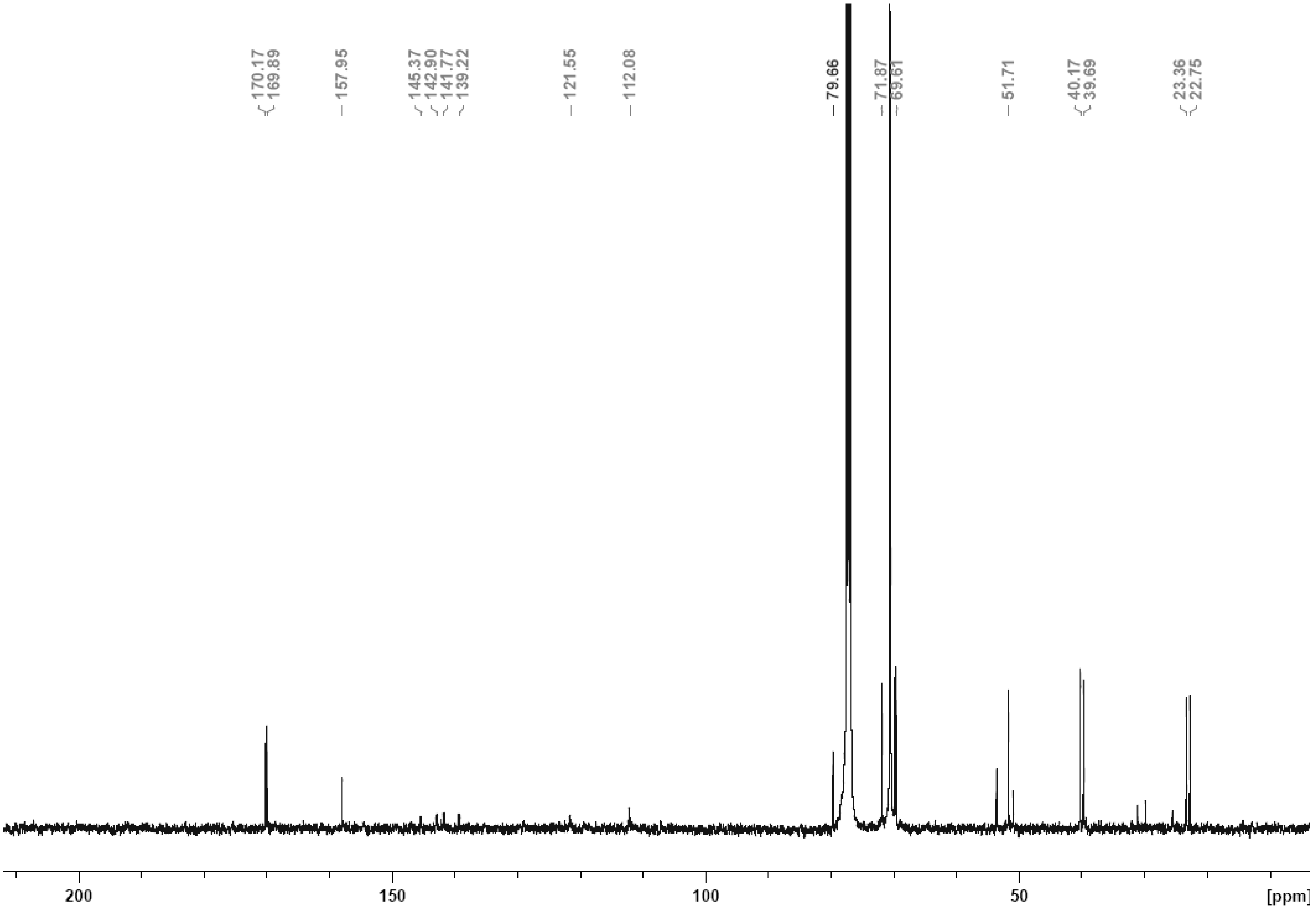

170.17
169.89
157.95
145.37
142.90
141.77
139.22
121.55
112.08
79.66
71.87
69.61
51.71
40.17
39.69
23.36
22.75
200
150
100
50
[ppm]

9.6. Aryl azide S9

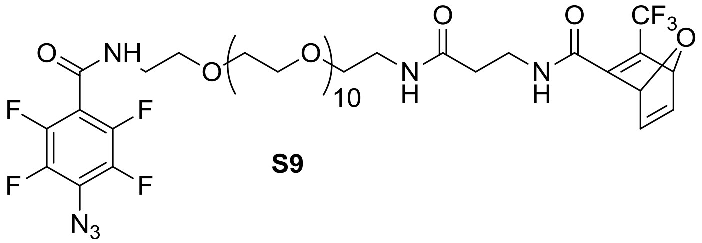

S9

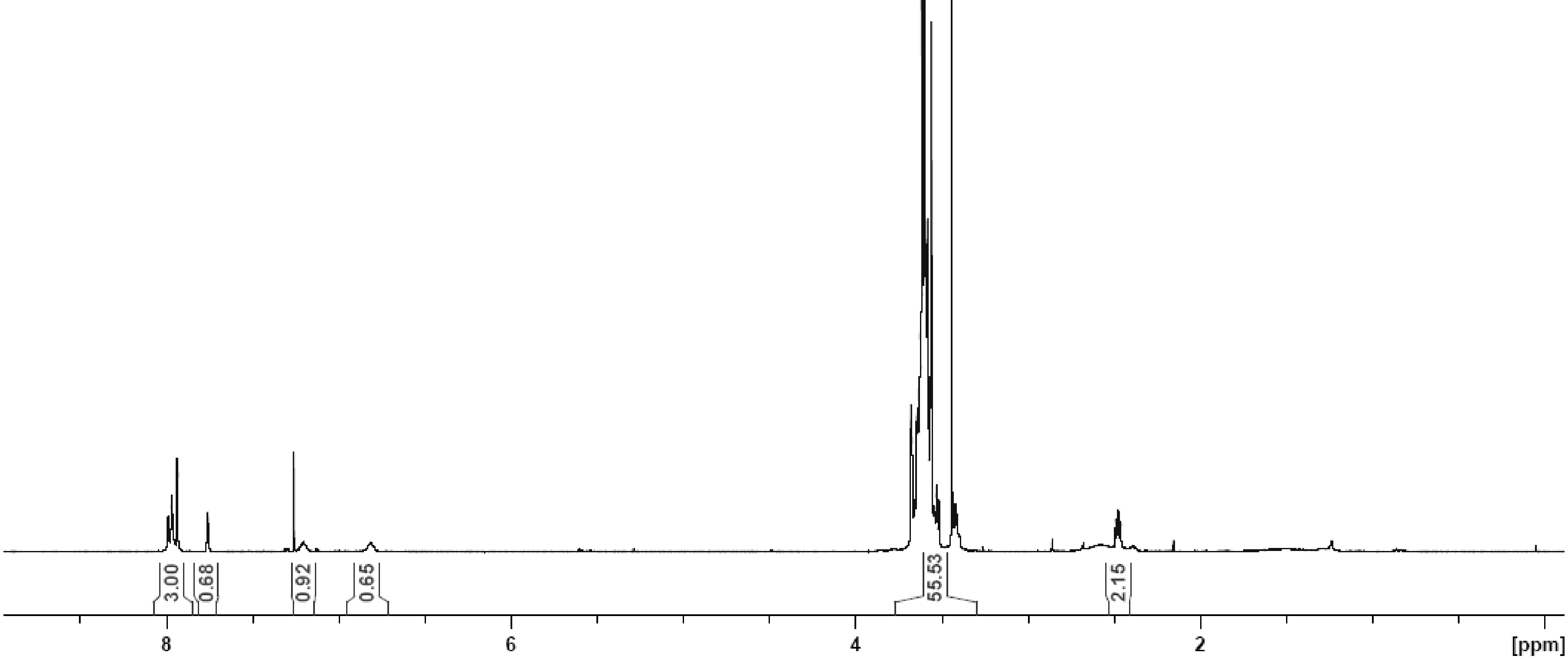

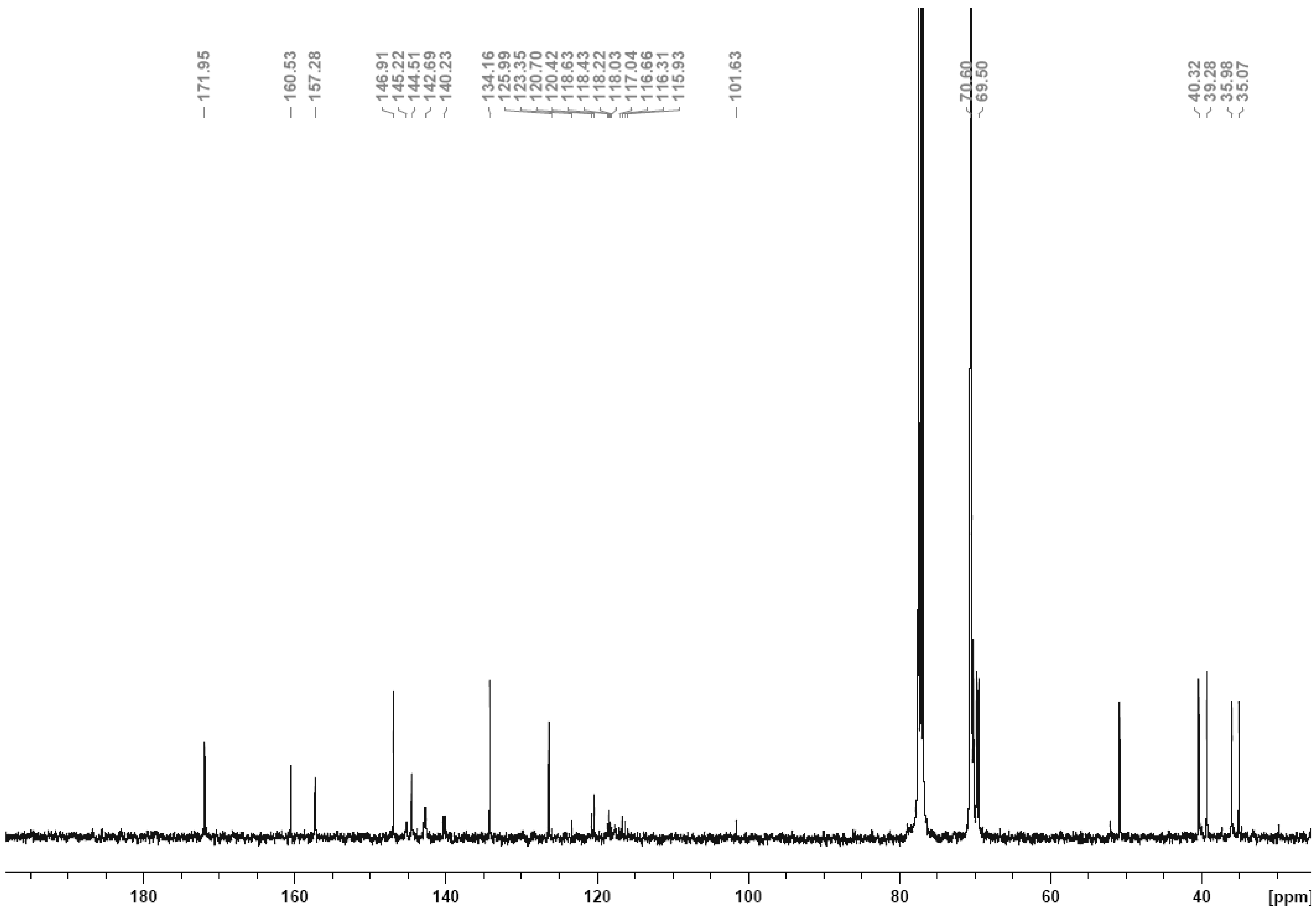

171.95
160.53
157.28
146.91
145.22
144.51
142.69
140.23
134.16
125.99
123.35
120.70
120.42
118.63
118.43
118.22
118.03
117.04
116.66
116.31
115.93
101.63
70.60
69.50
40.32
39.28
35.98
35.07
180
160
140
120
100
80
60
40
[ppm]

9.7. Fluorescein derivative S10

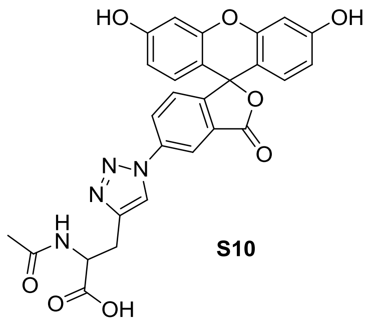

S10

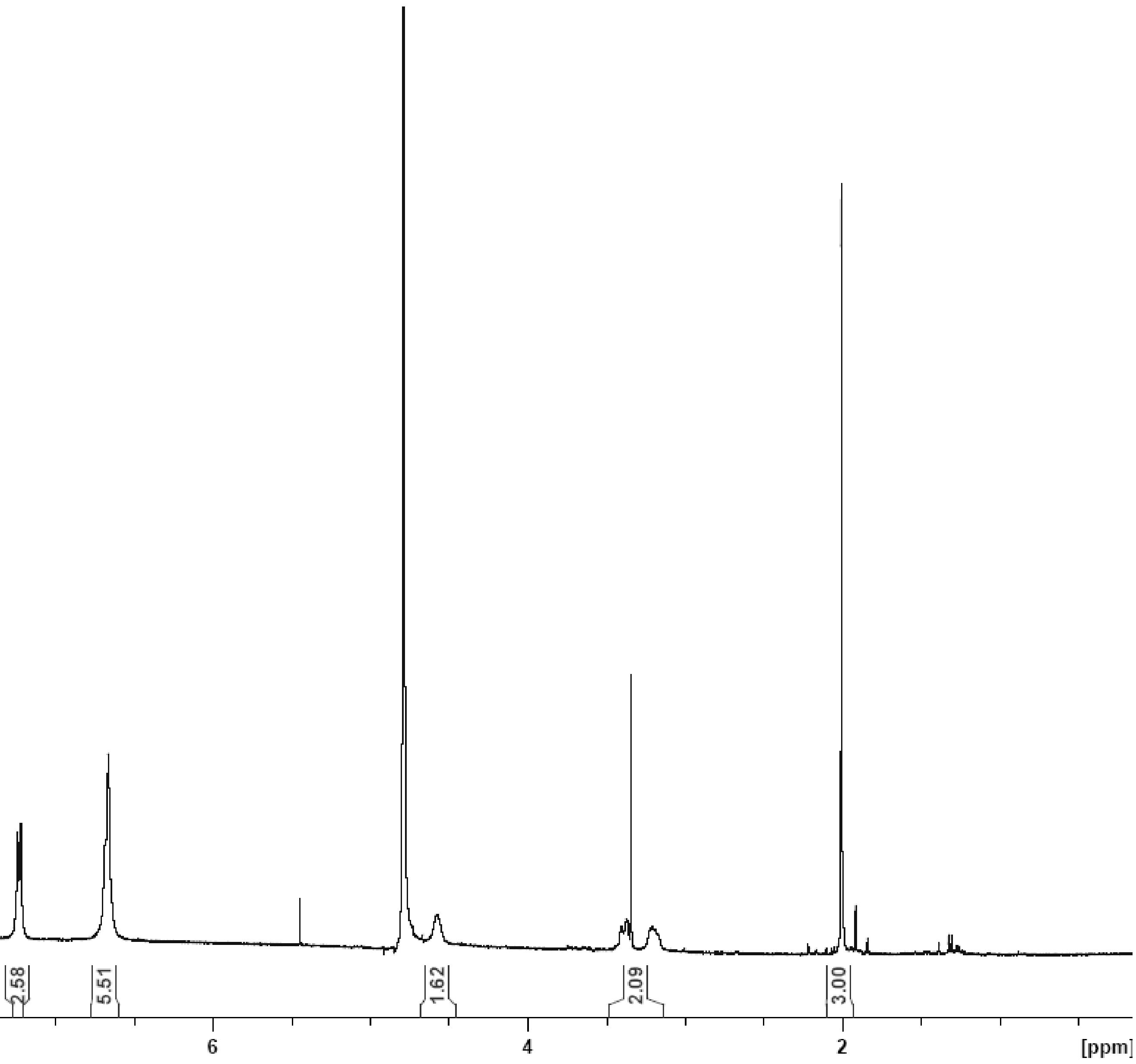

9.8. Triazole S13b

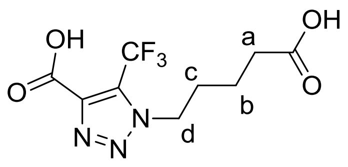

S13b

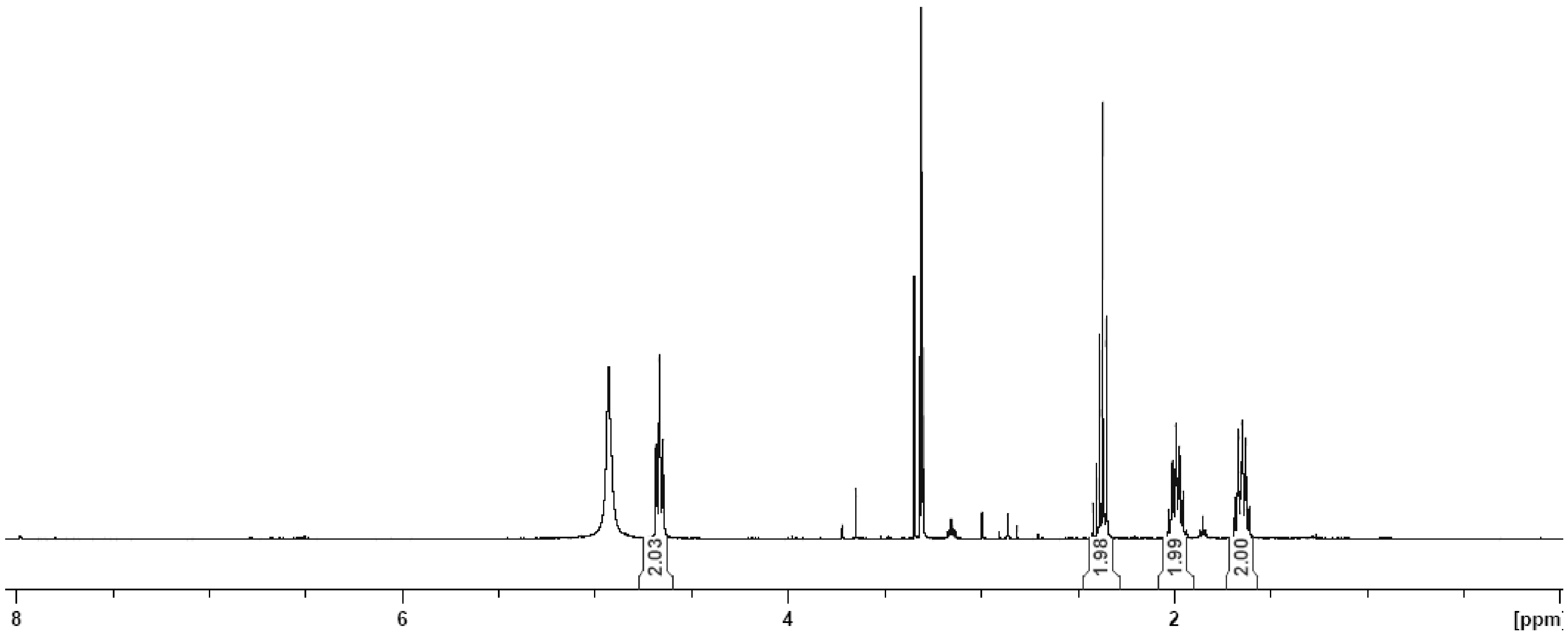

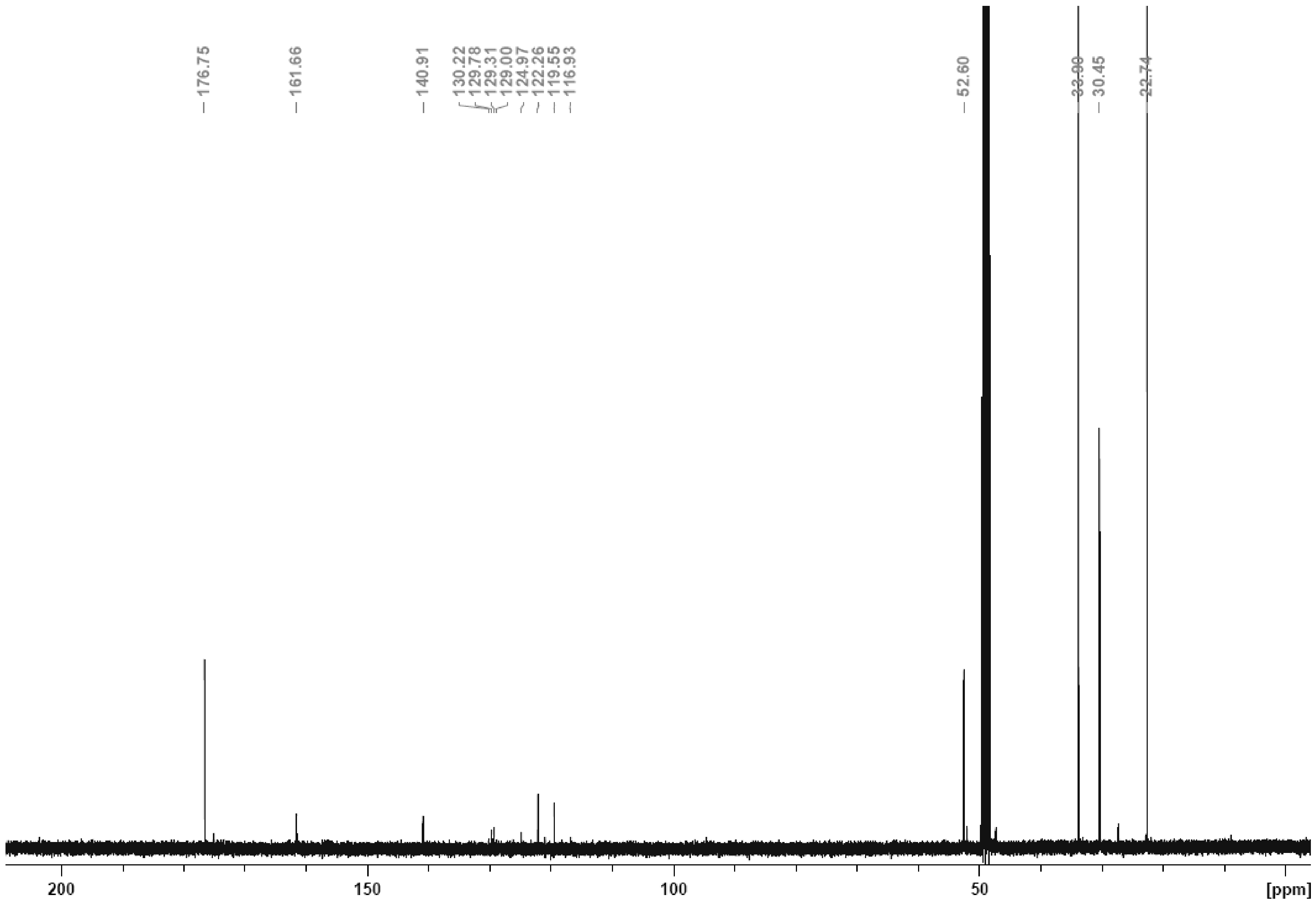

176.75
161.66
140.91
130.22
129.78
129.31
129.00
124.97
122.26
119.55
116.93
52.60
33.98
30.45
22.74
200
150
100
50
[ppm]

9.9. Triazole S13a

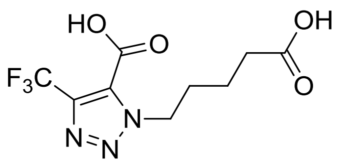

S13a

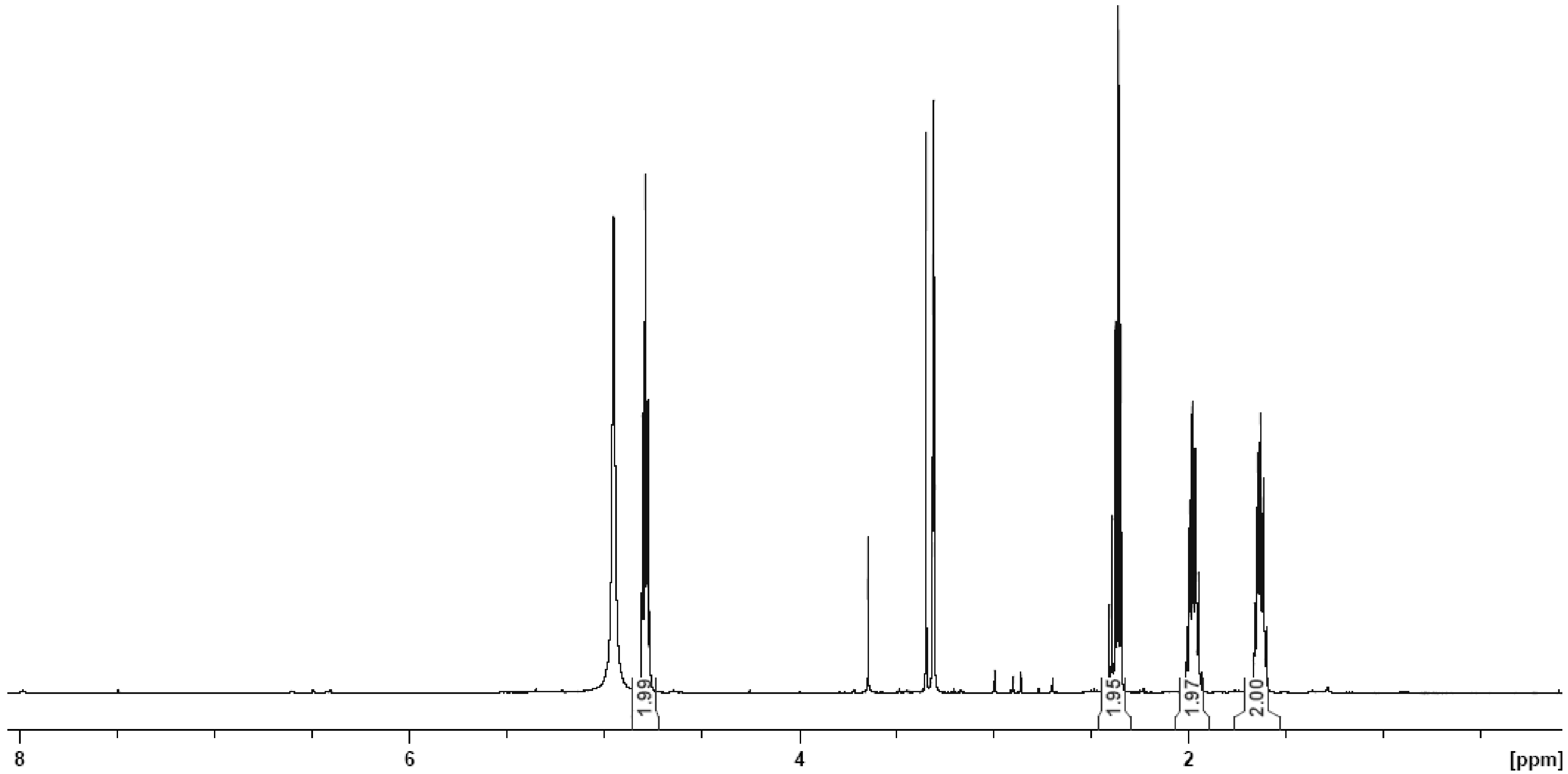

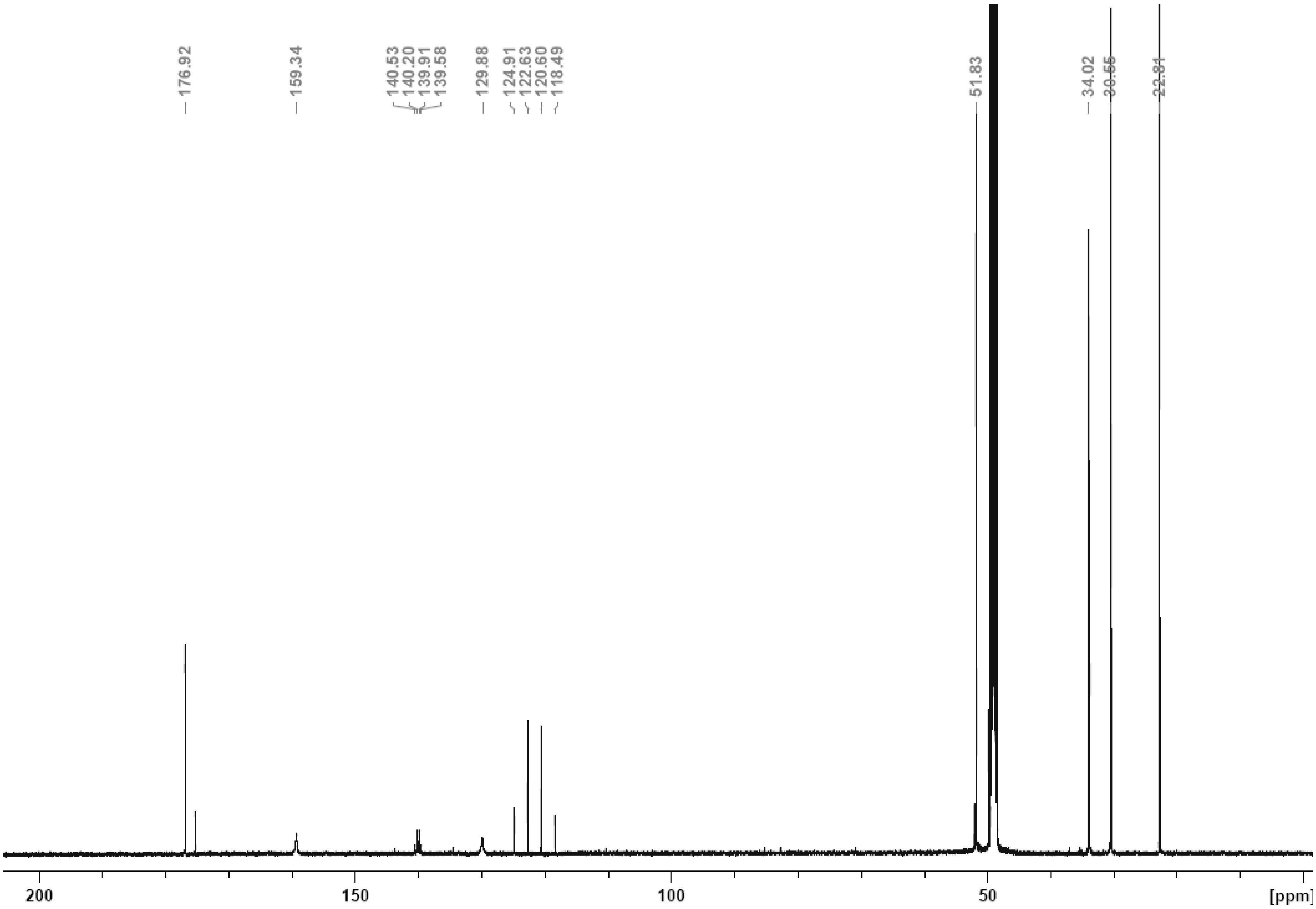
176.92
159.34
140.53
140.20
139.91
139.58
129.88
124.91
122.63
120.60
118.49
51.83
34.02
22.81
200
150
100
50
[ppm]

10. References

1. Keana, J. F. W.; Cai, S. X. *J. Org. Chem*. **1990**, *55*, 3640.

2. Brea, R. J.; Lopez-Deber, M. P.; Castedo, L.; Granja, J. R. *J. Org. Chem*. **2006**, *71*, 7870.

3. a) Rotman, A.; Heldman, J. *Biochemistry* **1981**, *20*, 5995; b) Molnar, E.; Varga, S.; Jona, I.; Martonosi, A. *Biochimica Biophysica Acta – Biomembranes* **1991**, *1068*, 27-40.

4. Su, Y.; Kasper,C.; Kirschning, A.; Dräger,G.; Berski, S. Macromol. Biosci. **2010**, *10*, 1028.

5. a) van Berkel, S. S.; Dirks, A. J.; Debets, M. F.; van Delft, F. L.; Cornelissen, J. J. L. M.; Nolte, R. J. M.; Rutjes, F. P. J., *ChemBioChem* **2007**, *8*, 1504–1508; b) van Berkel, S. S.; Dirks, A. J.; Meeuwissen, S. A.; Pingen, D. L. L.; Boerman, O. C.; Laverman, P.; van Delft, F. L.; Cornelissen, J. J. L. M.; Rutjes, F. P. J. T., *ChemBioChem* **2008**, *9*, 1805 – 1815; c) Kuijpers, B. H. M.; Groothuys, S. G. ; Soede, A. C.; Laverman, P.; Boerman, O. C.; van Delft, F. L.; Rutjes, F. P. J. T., *Bioconjugate Chem.* **2007**, *18*, 1847–1854.

6. Hess, C.; Wiegmann, B.; Maurer, A. N.; Fischer, P.; Möller, L.; Martin, U.; Hilfiker, A.; Haverich, A.; Fischer, S. *Tissue Engin., Part A*. **2010**, *16*, 3043-3053.
